# Lactate as a predictive biomarker and therapeutic target in cardiopulmonary bypass-associated acute kidney injury

**DOI:** 10.1016/j.isci.2026.117643

**Published:** 2026-10-08

**Authors:** Dinghao Xue, Mingru Zhang, Xinlu Chang, Tianlong Wang, Bingyang Ji, Chunhua Xi, Guyan Wang

**Affiliations:** 1 Department of Anesthesiology, Beijing Tongren Hospital, Capital Medical University, Beijing 100730, China; 2 Department of Cardiopulmonary Bypass, Fuwai Hospital, National Center for Cardiovascular Disease, State Key Laboratory of Cardiovascular Medicine, Chinese Academy of Medical Sciences & Peking Union Medical College, Beijing 100037, China

**Keywords:** cardiopulmonary bypass, acute kidney injury, machine learning, lactate, lactylation

## Abstract

Elevated lactate is a common metabolic perturbation following cardiopulmonary bypass (CPB). This study evaluates the clinical predictive value of lactate and explores its mechanism involving protein lactylation. Machine learning (ML) algorithms applied to multicenter intensive care databases identified preoperative lactate as a dominant predictor of CPB-acute kidney injury (AKI) risk. To validate these predictions biologically, rat CPB and cellular models were utilized. Experimental results demonstrated that CPB induces glycolytic reprogramming, lactate accumulation, and elevated pan-lysine lactylation. Inhibiting the production of lactate was associated with decreased protein lactylation, lower oxidative stress, and reduced local tissue inflammation, thereby mitigating renal injury. Collectively, our interpretable ML predictive models identify lactate as a top clinical predictor of CPB-AKI, while our experimental data establish the lactate-lactylation axis as a potential mechanistic pathway linking metabolic dysfunction to renal damage. This axis represents a candidate therapeutic target requiring further validation.

## Highlights

• ML identifies preoperative lactate as a top predictor of CPB-AKI risk
• CPB induces glycolytic reprogramming and renal lactate accumulation
• Accumulated lactate correlates with elevated global protein lactylation
• Inhibiting lactate production reduces lactylation and attenuates CPB-AKI

## Graphical abstract

**Figure:**
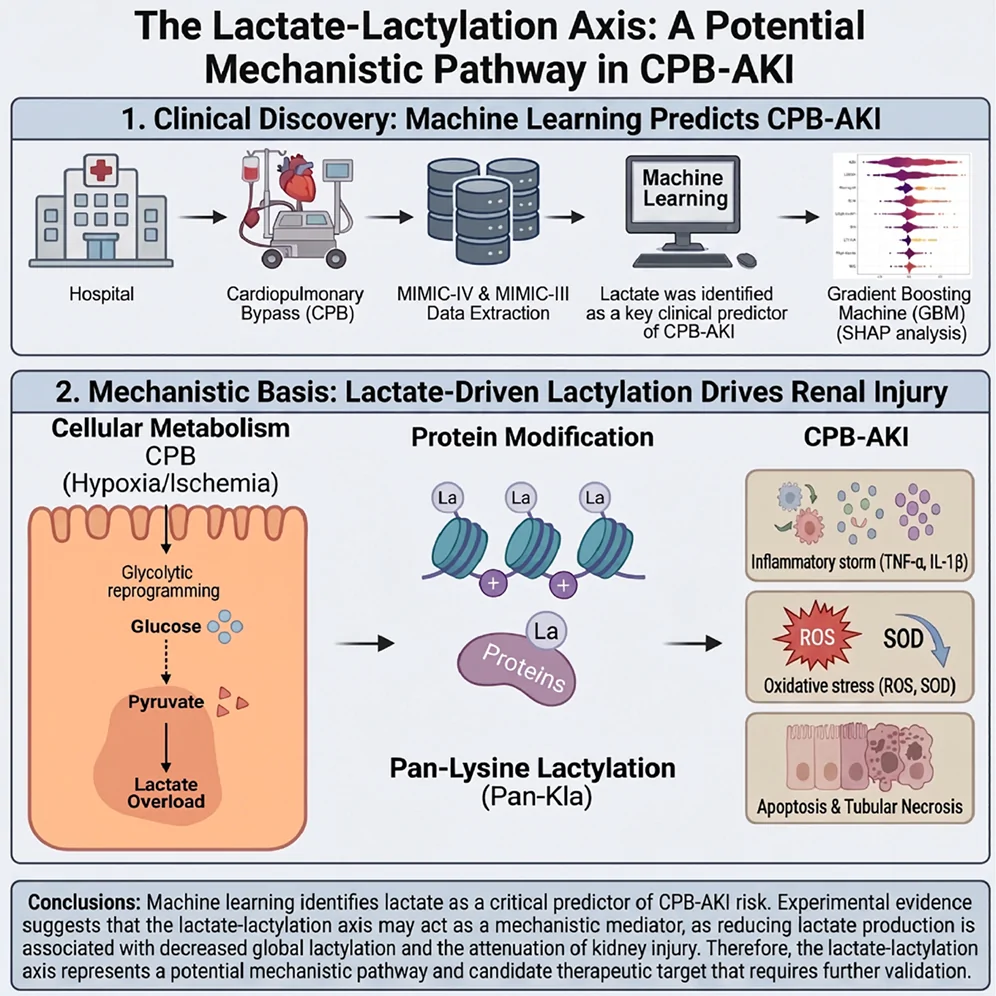

## Teaser

Health sciences

## Introduction

Cardiopulmonary bypass (CPB) serves as an indispensable core supportive technology in cardiovascular surgery. By substituting the physiological functions of the heart and lungs to maintain systemic oxygenation and circulatory homeostasis during operation, it creates an ideal bloodless and static surgical field for complex procedures—substantially advancing the development and efficacy of interventions such as coronary artery bypass grafting (CABG), heart valve replacement, and aortic aneurysm repair.^1^^,^^2^ However, multiple CPB-related pathophysiological perturbations, including hemodynamic disturbances, inflammatory responses, renal perfusion imbalance, and hemolysis, render acute kidney injury (AKI) one of the most prevalent and severe postoperative complications, with its incidence reaching 20.4%–40.5%.^3^^,^^4^^,^^5^ CPB-associated AKI (CPB-AKI) is closely linked to prolonged hospital stay, increased in-hospital mortality, and long-term chronic kidney disease (CKD) progression, imposing a substantial burden on healthcare systems worldwide.^6^^,^^7^ Despite advances in perioperative management and CPB technology, the clinical outcomes of CPB-AKI remain suboptimal, primarily due to the lack of reliable early prediction tools and incomplete understanding of its underlying pathogenic mechanisms.

Traditional clinical scoring systems often fall short in accurately predicting the risk of CPB-AKI. With the rapid advancement of artificial intelligence, interpretable machine learning (ML) models have emerged as promising tools for risk stratification in clinical practice, as they are able to integrate multiple variables to enhance predictive accuracy while clarifying the contribution of each individual factor.^8^^,^^9^^,^^10^ Among various clinical parameters, elevated blood lactate is a ubiquitous phenomenon during and after CPB.^11^^,^^12^ Traditionally, lactate has been regarded merely as a metabolic byproduct of anaerobic glycolysis and a passive “bystander” reflecting tissue hypoxia or hypoperfusion. However, emerging evidence suggests that lactate possesses active immunomodulatory and tissue-damaging properties.^13^^,^^14^ This raises a critical question: in the context of CPB-AKI, is lactate merely a predictive biomarker, or does it also contribute mechanistically to the progression of renal injury?

Recent advances in epigenetics have expanded our understanding of lactate’s biological role, revealing that it can serve as a direct donor for protein lactylation.^15^ This modification profoundly alters protein structure and function, actively participating in macrophage polarization, inflammatory storms, and organ injury.^16^^,^^17^ However, the role of lactylation in CPB-AKI and its upstream regulatory mechanisms have not been elucidated to date.

To bridge this gap, the present study employed a comprehensive “clinical-to-bench” approach. We first developed and validated ML models using the Medical Information Mart for Intensive Care (MIMIC)-IV and MIMIC-III databases to predict CPB-AKI risk, utilizing Shapley additive explanations (SHAP) analysis to identify lactate as a top clinical predictor. Furthermore, we established an *in vivo* rat CPB model and an *in vitro* oxygen-glucose deprivation/reoxygenation (OGD/R) model in a human renal proximal tubular epithelial cell line (HK-2). By integrating transcriptomic profiling with pharmacological inhibition and genetic silencing of lactate dehydrogenase A (LDHA), we aimed to elucidate the causal role of the lactate-driven lactylation axis in the pathogenesis of CPB-AKI. Our findings are expected to provide evidence that targeting lactate production and lactylation represents a promising therapeutic strategy for mitigating CPB-AKI.

## Results

### Baseline characteristics

A total of 6,558 patients who underwent CPB were included in the final analysis, comprising 3,613 patients from the MIMIC-IV database and 2,945 patients from the MIMIC-III database (Figure 1). The overall incidence of CPB-AKI was 74.20% (2,681/3,613) in the MIMIC-IV dataset and 91.82% (2,704/2,945) in the MIMIC-III dataset. Baseline characteristics between the MIMIC-IV and MIMIC-III cohorts are summarized in Table S1.

**Figure 1:**
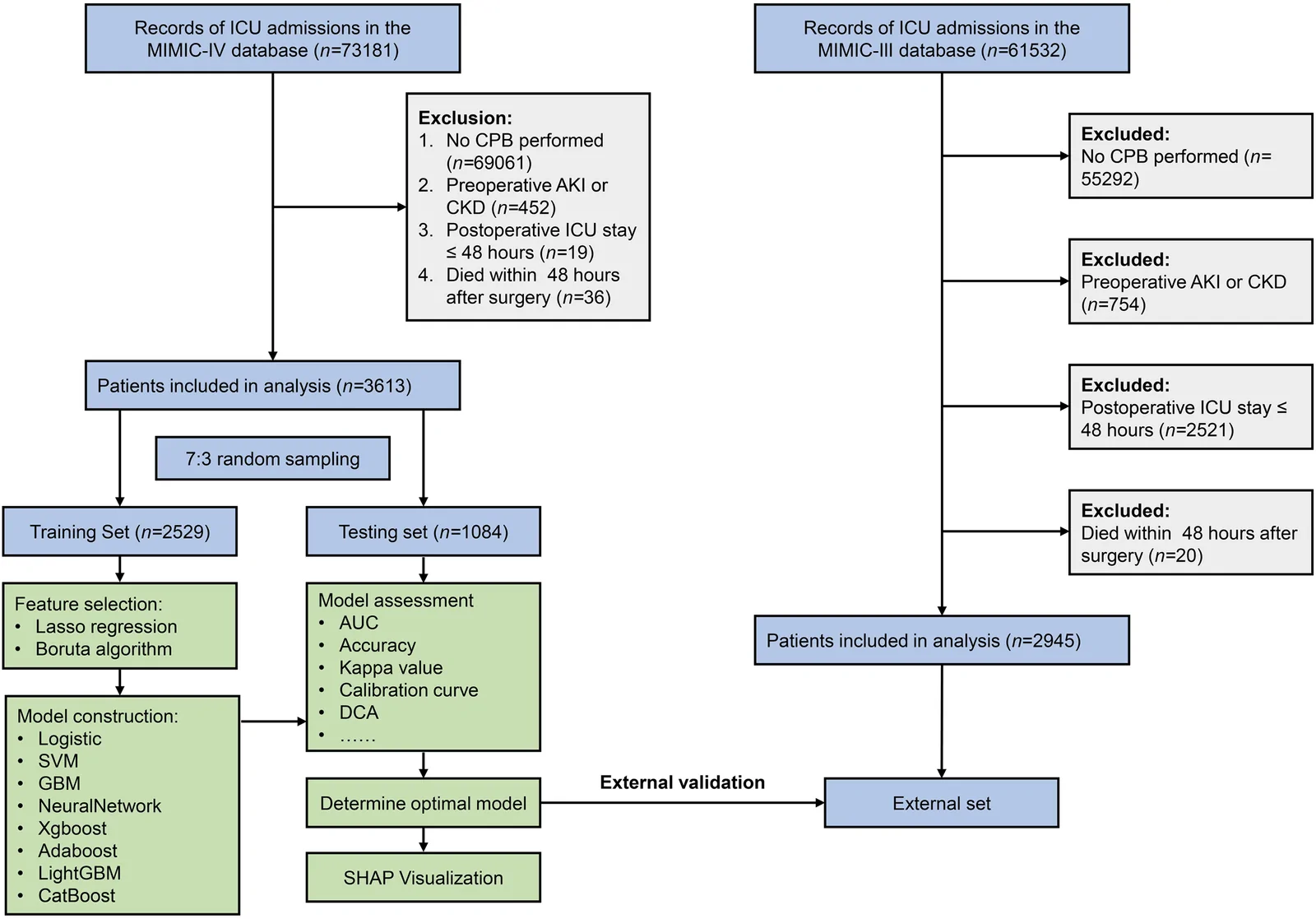
Study flow chart

The primary MIMIC-IV cohort was randomly partitioned into a training set (*n =* 2,529) and a testing set (*n =* 1,084) for model development and internal validation. Baseline characteristics were well-balanced between these two sets, with no statistically significant differences in most demographics, comorbidities (e.g., hypertension, coronary heart disease [CHD], and heart failure), or laboratory parameters (e.g., lactate, hemoglobin, and platelets). The incidence of CPB-AKI was 73.98% (*n =* 1,871) in the training set and 74.72% (*n =* 810) in the testing set, confirming the comparability of the datasets for ML applications (Table 1). Furthermore, patients who developed CPB-AKI were significantly older, had a higher prevalence of comorbidities, and exhibited elevated preoperative lactate levels compared to those without CPB-AKI (Table S2).

**Table 1:** Baseline characteristics of patients in the training and testing sets in the MIMIC-IV dataset

Characteristics	Overall (*n* = 3,613)	Training set (*n* = 2,529)	Testing set (*n* = 1,084)	*p* value
Age, years (median [Q1, Q3])	68 (60–76)	68 (61–76)	68 (60–76.25)	0.463
Gender, *n* (%)				
Female	2,520 (69.75)	1,763 (69.71)	757 (69.83)	0.973
Male	1,093 (30.25)	766 (30.29)	327 (30.17)	
BMI, kg/m^2^ (median [Q1, Q3])	29.245 (25.97–33.145)	29.306 (26.044–33.218)	29.144 (25.738–32.954)	0.222
Smoking history, *n* (%)				
No	2368 (65.54)	1650 (65.24)	718 (66.24)	0.591
Yes	1245 (34.46)	879 (34.76)	366 (33.76)	
Drinking history, *n* (%)				
No	3,509 (97.12)	2,450 (96.88)	1,059 (97.69)	0.216
Yes	104 (2.88)	79 (3.12)	25 (2.31)	
Hypertension, *n* (%)				
No	1,266 (35.04)	870 (34.40)	396 (36.53)	0.233
Yes	2,347 (64.96)	1,659 (65.60)	688 (63.47)	
Diabetes mellitus, *n* (%)				
No	2527 (69.94)	1734 (68.56)	793 (73.15)	0.007
Yes	1086 (30.06)	795 (31.44)	291 (26.85)	
CHD, *n* (%)				
No	987 (27.32)	681 (26.93)	306 (28.23)	0.445
Yes	2,626 (72.68)	1,848 (73.07)	778 (71.77)	
Heart failure, *n* (%)				
No	2,828 (78.27)	1,989 (78.65)	839 (77.40)	0.429
Yes	785 (21.73)	540 (21.35)	245 (22.60)	
CVA, *n* (%)				
No	3,333 (92.25)	2,328 (92.05)	1,005 (92.71)	0.541
Yes	280 (7.75)	201 (7.95)	79 (7.29)	
COPD, *n* (%)				
No	3,312 (91.67)	2,317 (91.62)	995 (91.79)	0.915
Yes	301 (8.33)	212 (8.38)	89 (8.21)	
SOFA score (median [Q1, Q3])	5 (3–7)	5 (3–7)	5 (3–7)	0.021
SAPS Ⅱ (median [Q1, Q3])	35 (28–42)	35 (28–43)	34.5 (28–42)	0.095
Lactate, mmol/L (median [Q1, Q3])	2.1 (1.6–2.8)	2.1 (1.6–2.8)	2.1 (1.6–2.725)	0.296
Hemoglobin, g/dL (median [Q1, Q3])	11.3 (9.7–13.1)	11.4 (9.7–13.2)	11.2 (9.5–13)	0.057
Platelet, ×10^9^/L (median [Q1, Q3])	180 (140–226)	179 (140–226)	181 (142–229)	0.211
RBC, ×10^12^/L (median [Q1, Q3])	3.75 (3.16–4.33)	3.77 (3.17–4.35)	3.705 (3.117–4.272)	0.038
WBC, ×10^9^/L (median [Q1, Q3])	9.4 (7.1–13)	9.4 (7.1–12.9)	9.5 (7.175–13.2)	0.238
Chloride, mmol/L (median [Q1, Q3])	107 (103–111)	107 (103–110)	107 (103–111)	0.269
Glucose, mg/dL (median [Q1, Q3])	115 (100–134)	116 (101–134)	114 (100–132)	0.034
Potassium, mmol/L (median [Q1, Q3])	4.1 (3.9–4.4)	4.1 (3.9–4.4)	4.1 (3.9–4.4)	0.379
Sodium, mmol/L (median [Q1, Q3])	140 (138–141)	140 (138–141)	140 (138–141)	0.805
PaCO_2_, mmHg (median [Q1, Q3])	41 (38–45)	41 (38–45)	41 (38–44)	0.521
PaO_2_, mmHg (median [Q1, Q3])	380 (311–429)	378 (306–429)	383 (318–429)	0.149
INR (median [Q1, Q3])	1.2 (1.1–1.4)	1.2 (1.1–1.4)	1.2 (1.1–1.4)	0.780
PT, s (median [Q1, Q3])	13.8 (12.3–15.4)	13.8 (12.3–15.3)	13.9 (12.4–15.4)	0.911
Scr, mg/dL (median [Q1, Q3])	0.9 (0.8–1.1)	0.9 (0.8–1.1)	0.9 (0.8–1.1)	0.896
BUN, mg/dL (median [Q1, Q3])	17 (13–21)	17 (13–21)	17 (14–21)	0.568
Type of surgery, *n* (%)				
CABG	1,736 (48.05)	1,221 (48.28)	515 (47.51)	0.317
Valve surgery	1,072 (29.67)	744 (29.42)	328 (30.26)	
Aortic surgery	16 (0.44)	15 (0.59)	1 (0.09)	
Combined surgery	114 (3.16)	78 (3.08)	36 (3.32)	
Others	675 (18.68)	471 (18.62)	204 (18.82)	
CPB-AKI, *n* (%)				
No	932 (25.80)	658 (26.02)	274 (25.28)	0.671
Yes	2,681 (74.20)	1,871 (73.98)	810 (74.72)	

BMI, body mass index; CHD, coronary heart disease; CVA, cerebrovascular accident; COPD, chronic obstructive pulmonary disease; SOFA, sequential organ failure assessment; SAPS Ⅱ, simplified acute physiology score Ⅱ; RBC, red blood cell; WBC, white blood cell; PaCO_2_, partial pressure of carbon dioxide; PaO_2_, partial pressure of oxygen; INR, international normalized ratio; PT, prothrombin time; Scr, serum creatinine; BUN, blood urea nitrogen; CABG, coronary artery bypass grafting; CPB-AKI, cardiopulmonary bypass-associated acute kidney injury. See also Tables S1 and S2.

### Feature selection and model development

A dual feature selection strategy was employed on the training set. The Boruta algorithm was applied to evaluate feature importance, identifying 27 variables associated with CPB-AKI (Figures 2A and 2B). Concurrently, least absolute shrinkage and selection operator (LASSO) regression, optimized via 10-fold cross-validation, was utilized for feature shrinkage. The misclassification errors against log(lambda) and the LASSO regression coefficient profiles are shown in Figures 2C and 2D. The intersection of the features selected by Boruta and LASSO yielded a consensus subset of nine variables (Figure 2E). These overlapping features—age, body mass index (BMI), lactate, simplified acute physiology score II (SAPS II), potassium, serum creatinine (Scr), surgery type, blood urea nitrogen (BUN), and heart failure—were subsequently utilized for the construction of predictive models.

**Figure 2:**
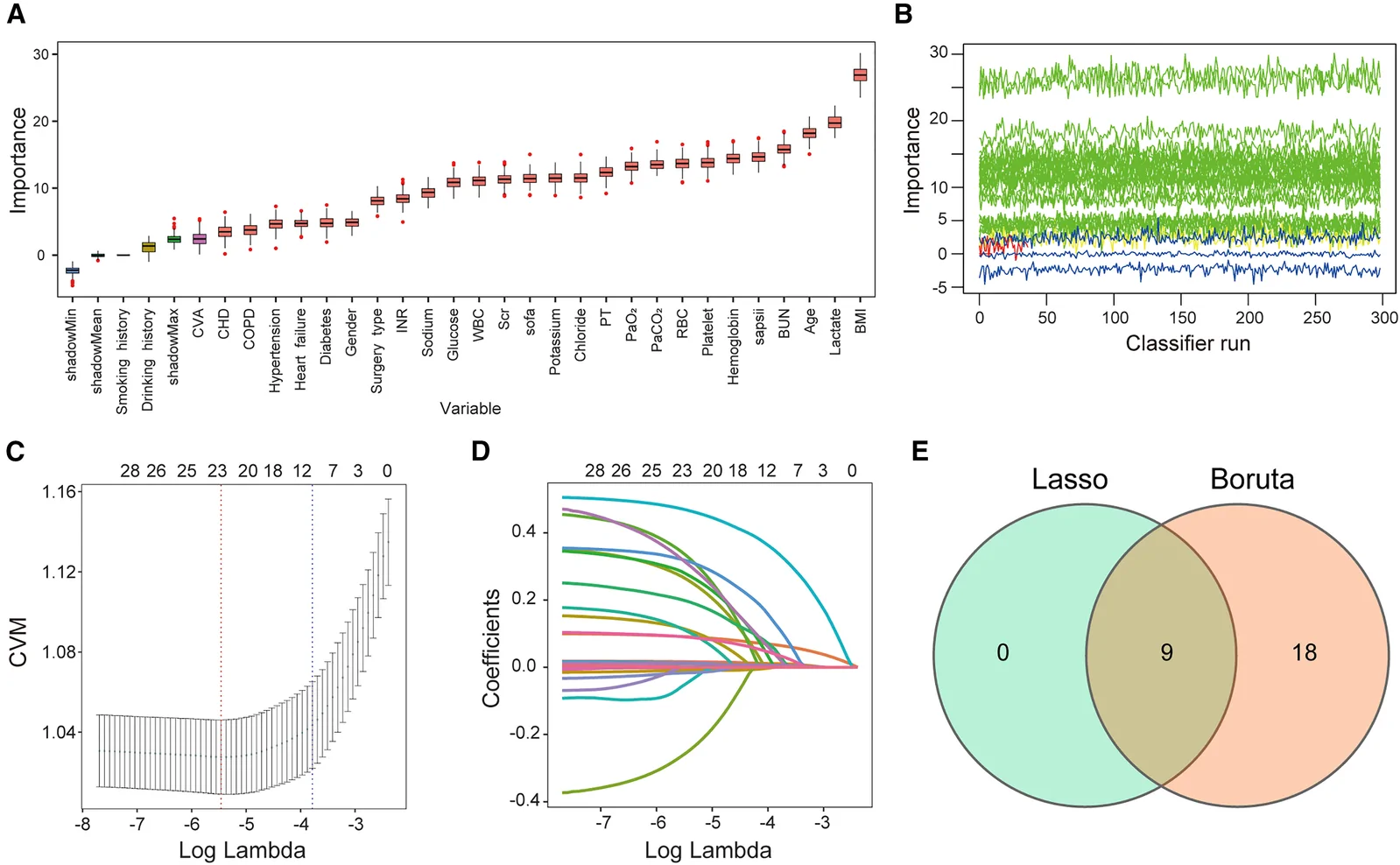
Predictor screening results (A and B) Feature selection using the Boruta algorithm. (A) Boxplots illustrating the importance distribution of candidate variables. (B) The trajectory of variable importance scores across 300 classifier runs. (C) 10-fold cross-validation for parameter tuning in the LASSO regression model. The cross-validation mean (CVM) is plotted against log(λ). The two vertical dashed lines indicate the optimal λ values based on the minimum criterion (left) and the 1-standard error (1-SE) criterion (right). (D) LASSO coefficient profiles of the candidate variables. Each curve represents the trajectory of a single variable’s coefficient as the tuning parameter log(λ) varies. (E) Venn diagram illustrating the intersection of features independently selected by the LASSO and Boruta algorithms, yielding 9 consistently important consensus predictors.

### Model performance and validation

To evaluate predictive capabilities for CPB-AKI, eight ML models, gradient boosting machine (GBM), LightGBM, logistic regression, category boosting (CatBoost), support vector machine (SVM), extreme gradient boosting (XGBoost), neural network, and adaptive boosting (AdaBoost), were constructed and compared using the testing set. The GBM model demonstrated the highest performance across multiple evaluation metrics, achieving an accuracy of 0.834, sensitivity of 0.817, specificity of 0.884, precision of 0.956, F1 score of 0.881, and kappa value of 0.611 (Table 2). Receiver operating characteristic (ROC) curve analysis showed that the GBM model yielded the highest area under the ROC (AUC) of 0.898 (95% confidence interval [CI]: 0.876–0.919) in the internal testing set, followed by the LightGBM (AUC: 0.840; 95% CI: 0.815–0.866) and CatBoost (AUC: 0.783; 95% CI: 0.755–0.812) models (Figure 3A).

**Table 2:** Predictive performance of eight machine learning models for CPB-AKI in the testing set

Model	Accuracy	Sensitivity	Specificity	Precision	F1 score	Kappa value
GBM	0.834	0.817	0.884	0.956	0.881	0.6114
LightGBM	0.781	0.789	0.757	0.908	0.845	0.4821
Logistic regression	0.672	0.674	0.664	0.859	0.755	0.2771
CatBoost	0.668	0.613	0.836	0.919	0.735	0.3346
SVM	0.637	0.614	0.705	0.864	0.718	0.2463
XGBoost	0.631	0.574	0.806	0.900	0.701	0.2769
Neural network	0.616	0.545	0.832	0.908	0.681	0.2680
AdaBoost	0.570	0.493	0.806	0.886	0.633	0.2054

GBM, gradient boosting machine; LightGBM, light gradient boosting machine; CatBoost, category boosting; SVM, support vector machine; XGBoost, extreme gradient boosting; AdaBoost, adaptive boosting. See also Table S3.

**Figure 3:**
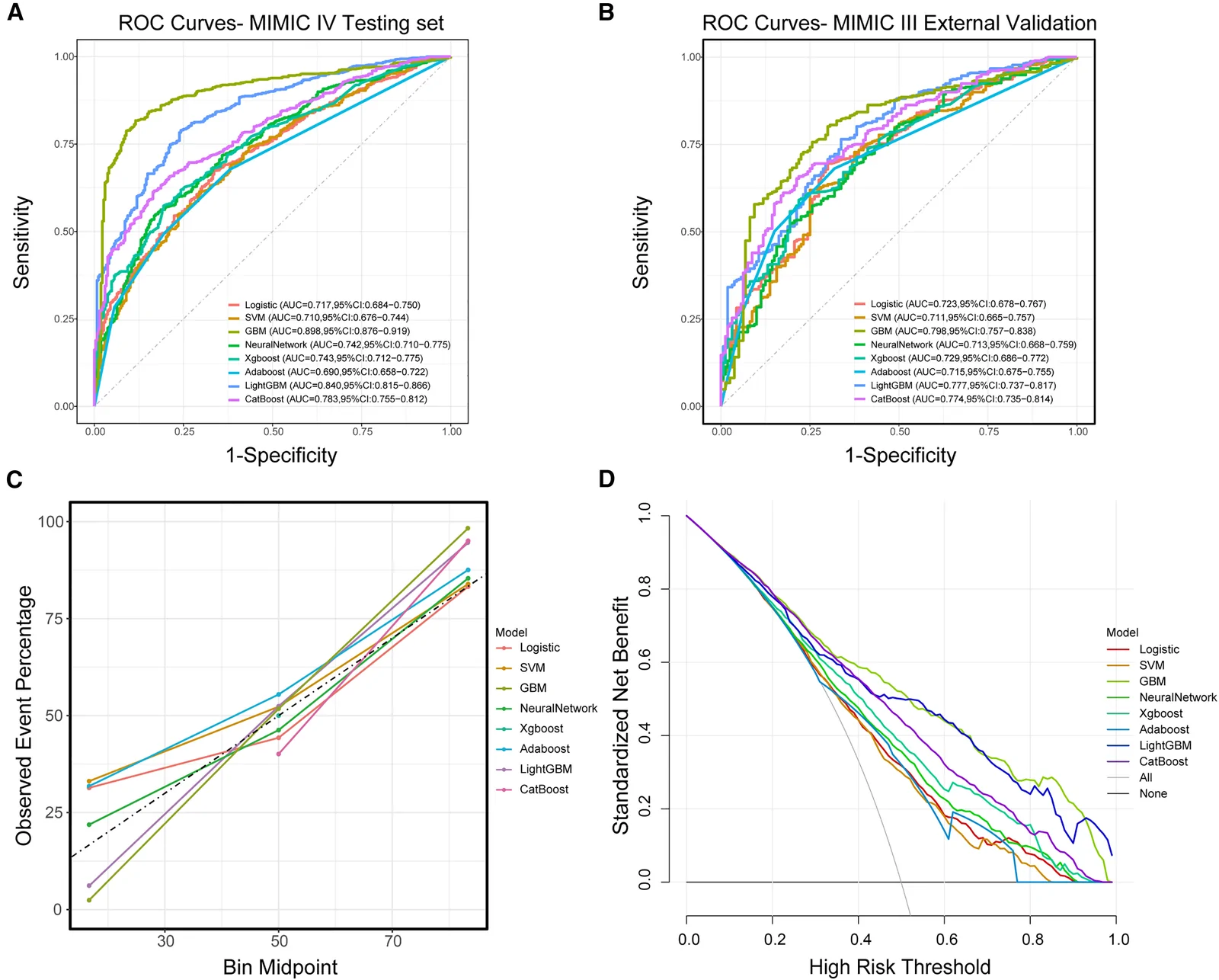
Machine learning-based models used to predict CPB-AKI (A and B) ROC curves comparing the discriminative performance of the eight predictive models in the MIMIC-IV testing set (A) and the MIMIC-III external validation set (B). The corresponding AUC values and 95% CI are detailed in the legends. (C) Calibration curves of the machine learning-based models. (D) DCA of the machine learning-based models.

External validation using the independent MIMIC-III dataset was conducted to assess model generalizability. The GBM model maintained its performance in the external cohort, achieving an AUC of 0.798 (95% CI: 0.757–0.838) (Figure 3B). Calibration curves indicated a high concordance between the predicted probabilities of the GBM model and the actual observed frequencies of CPB-AKI (Figure 3C). Furthermore, decision curve analysis (DCA) demonstrated that the GBM model provided a higher standardized net benefit across a wide range of threshold probabilities compared to the other models, suggesting its utility for guiding clinical decision-making (Figure 3D).

### Model interpretability and feature contributions via SHAP analysis

To elucidate the decision-making process of the GBM model, the SHAP framework was employed. Global feature importance was evaluated using mean absolute SHAP values (Figure S1A), identifying BMI, lactate, potassium, BUN, and surgery type as the top contributors to the model’s predictions.

The directional impact of these variables was assessed via a SHAP beeswarm plot. High feature values for BMI, lactate, potassium, BUN, and SAPS II correlated with positive SHAP values, indicating an association with increased CPB-AKI risk (Figure S1B). SHAP dependence plots illustrated the non-linear marginal effects of individual features (Figures 4A–4I). Specifically, the dependence plot for lactate demonstrated a progressive increase in the predicted risk of AKI corresponding to rising blood lactate concentrations.

**Figure 4:**
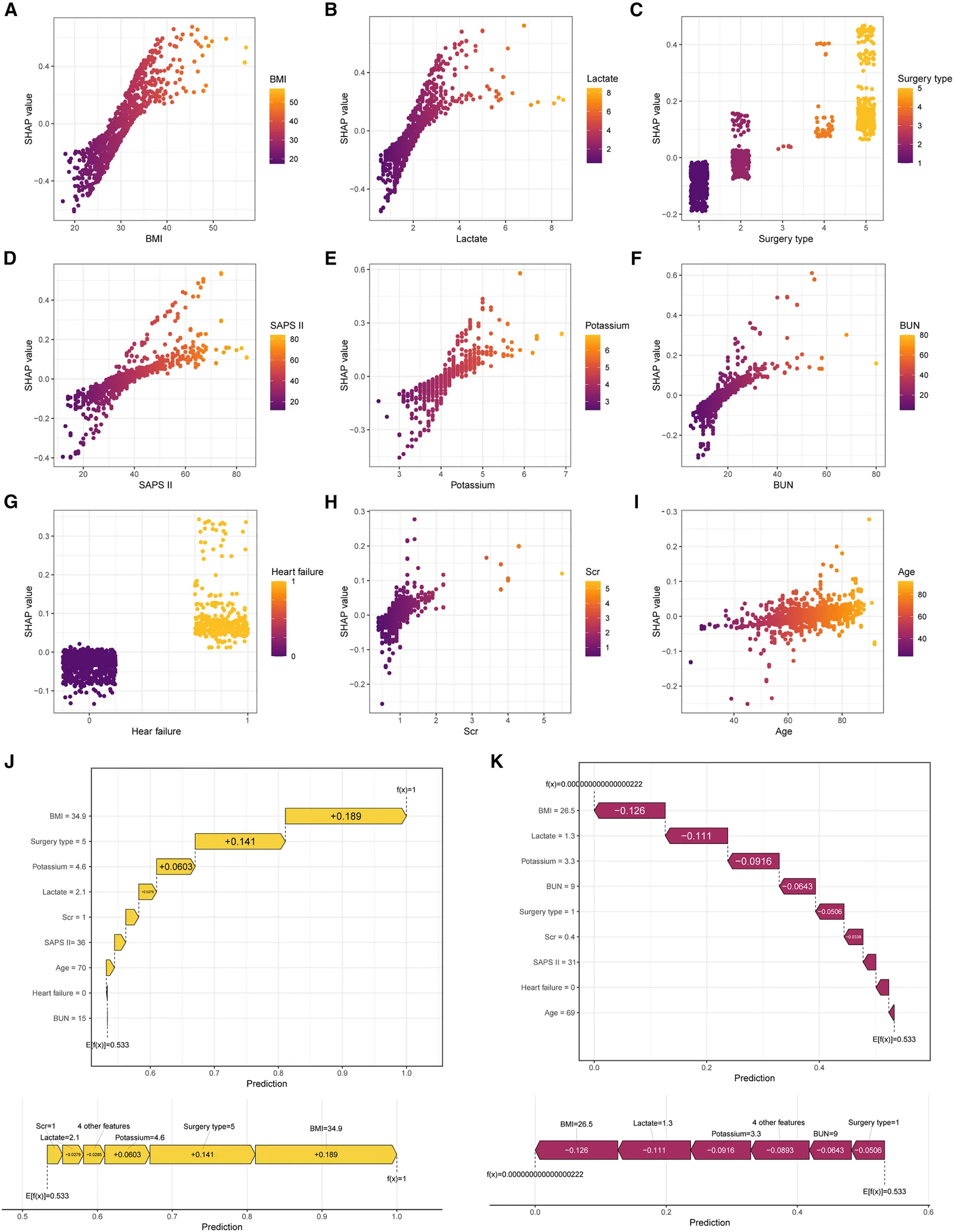
Global and local interpretability of the optimal predictive model via SHAP analysis (A–I) SHAP dependence plots illustrating the global impact, directionality, and non-linear effects of the top 9 critical predictors on the model output. For surgery type (C), numerical categories denote the following: 1, CABG; 2, valve surgery; 3, aortic surgery; 4, combined surgery; and 5, others. For heart failure (G), 0 indicates absence, and 1 indicates presence. (J and K) Waterfall plots (top) and corresponding force plots (bottom) demonstrating local interpretability for a representative high-risk patient (J) and a low-risk patient (K). These plots delineate how individualized feature attributions incrementally shift the specific prediction f(x) away from the baseline expected value E[f(x)]. See also Figure S1.

SHAP force plots were generated to illustrate individual risk predictions (Figures 4J and 4K). These plots delineate how specific patient characteristics contribute to the model’s output; for instance, a lactate level of 2.1 mmol/L and a BMI of 34.9 increased the risk probability by +0.0279 and +0.189, respectively. Lower physiological values correspondingly shifted the prediction toward a lower risk threshold. The identification of lactate as a top predictive feature provided the rationale for subsequent *in vivo* and *in vitro* mechanistic investigations into the lactate-lactylation axis.

### CPB induces AKI, lactate accumulation, and elevated lactylation *in vivo*

To investigate the biological role of lactate, a rat model of CPB was established (Figures 5A and S2). Blood lactate levels were monitored throughout the perioperative period. Compared to the sham group, rats in the CPB group exhibited increased blood lactate at the end of CPB (T1) and at 24 h post-operation (Figure 5B). Correspondingly, lactate concentrations within kidney tissues were elevated at 24 h post-CPB (Figure 5C).

**Figure 5:**
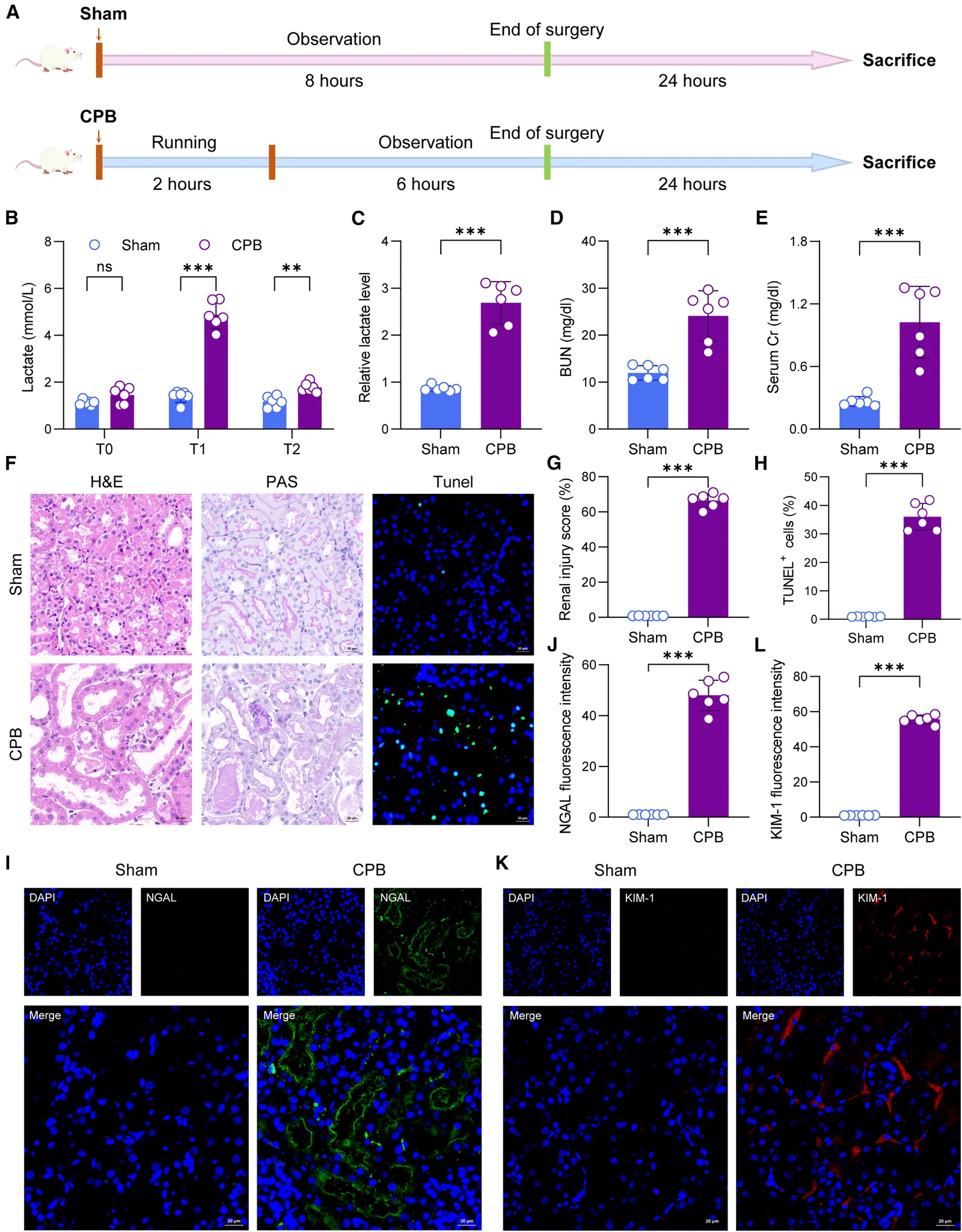
CPB induces severe acute kidney injury and concurrent lactate accumulation *in vivo* (A) Schematic timeline of the experimental design for the sham and CPB rat models. (B) Dynamic trajectory of arterial blood lactate concentrations across three critical time points: 10 min after CPB initiation (T0), immediately at the end of CPB (T1), and 24 h post-surgery (T2). *n =* 6; mean ± SD; one-way ANOVA with Tukey’s test. (C) Relative lactate levels quantified in renal tissues harvested at 24 h post-operation. *n =* 6; mean ± SD; unpaired *t* test. (D and E) Serum concentrations of BUN (D) and Scr (E) assessed at 24 h post-operation to evaluate renal function impairment. *n =* 6; mean ± SD; unpaired *t* test. (F) Representative histological images of H&E staining, PAS staining, and TUNEL fluorescence in renal tissues. Scale bars, 20 μm. (G and H) Semi-quantitative analysis of the renal tubular injury score (G) and the percentage of TUNEL-positive apoptotic cells (H). *n =* 6; mean ± SD; unpaired *t* test. (I–L) Representative immunofluorescence images and corresponding quantitative fluorescence intensity analysis of the early tubular injury markers NGAL (I and J) and KIM-1 (K and L) in renal sections. Nuclei were counterstained with DAPI (blue). Scale bars, 20 μm. *n =* 6; mean ± SD; unpaired *t* test. ∗∗*p* < 0.01, ∗∗∗*p* < 0.001; ns, not significant.

Renal functional assessment revealed increased BUN and Scr levels in the CPB group at 24 h post-operation (Figures 5D and 5E). Hematoxylin and eosin (H&E) staining and periodic acid-Schiff (PAS) staining showed tubular structural disruption, brush border loss, and cellular necrosis in the CPB group, accompanied by higher tubular injury scores (Figures 5F and 5G). Terminal deoxynucleotidyl transferase-mediated dUTP nick end labeling (TUNEL) staining indicated an increase in renal cell apoptosis following CPB (Figures 5F and 5H). Immunofluorescence intensity for the AKI biomarkers NGAL and KIM-1 was correspondingly increased in the renal tissues of CPB rats (Figures 5I–5L).

To determine whether lactate accumulation is associated with epigenetic modifications in injured kidneys, pan-lysine lactylation (Pan-Kla) levels were evaluated. Western blotting analysis showed increased Pan-Kla levels in the renal tissues of the CPB group compared to the sham group (Figures 6A and 6B). Immunofluorescence staining confirmed these findings, with Pan-Kla signals localized primarily within the renal tubular epithelial cells of CPB rats (Figures 6C and 6D). These results indicate that CPB induces AKI, accompanied by lactate accumulation and elevated protein lactylation *in vivo*.

**Figure 6:**
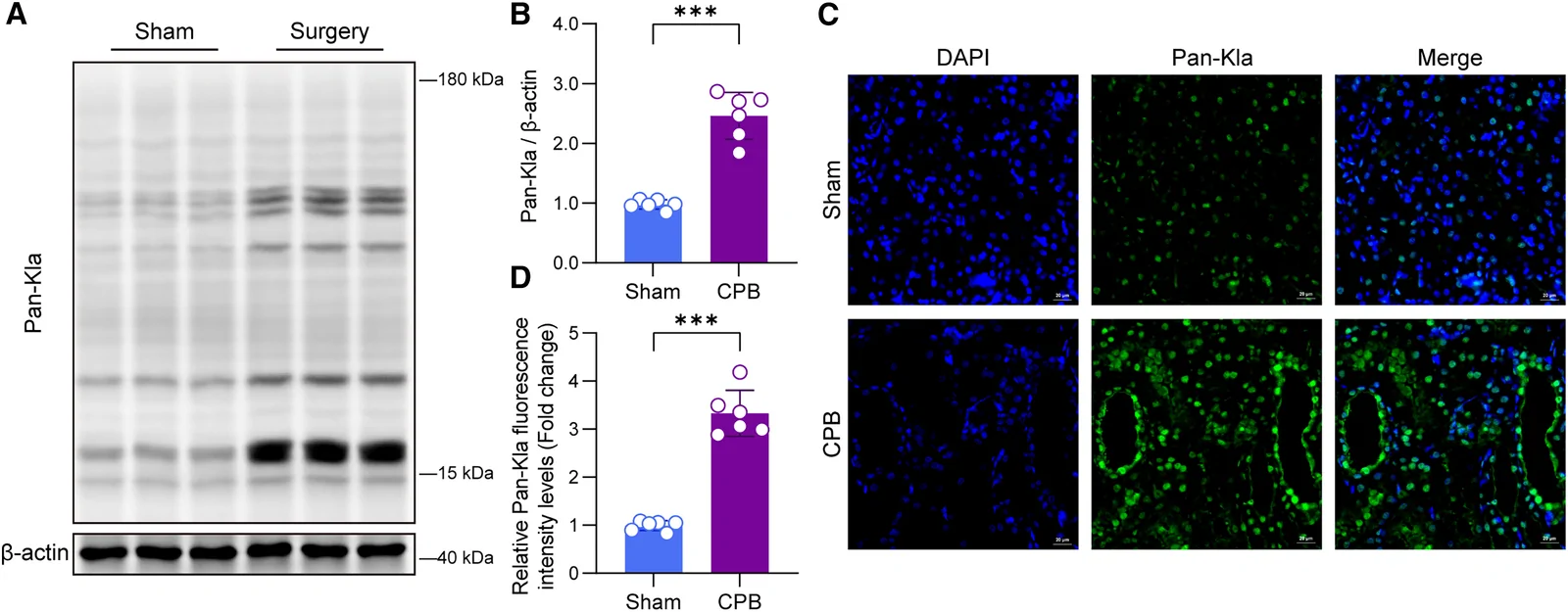
CPB upregulates global pan-lysine lactylation in renal tissues and tubular epithelial cells (A and B) Representative western blotting images (A) and corresponding densitometric quantification (B) of Pan-Kla levels in whole kidney tissue lysates. *n =* 6; mean ± SD; unpaired *t* test. (C and D) Representative immunofluorescence images (C) and quantitative fluorescence intensity analysis (D) demonstrating the spatial localization and significant upregulation of Pan-Kla (green) specifically within renal tubular epithelial cells. Nuclei were counterstained with DAPI (blue). Scale bars, 20 μm. *n =* 6; mean ± SD; unpaired *t* test. ∗∗∗*p* < 0.001.

### Transcriptomic profiling reveals distinct genome-wide expression signatures in CPB-induced AKI

To explore the molecular mechanisms of CPB-AKI, RNA sequencing (RNA-seq) was performed on renal tissues from the sham and CPB groups. Transcriptomic profiling identified distinct genome-wide gene expression patterns between the groups. The overall distribution of normalized gene expression levels was consistent across samples (Figures 7A and 7B). Pearson correlation analysis of the expression profiles indicated intra-group consistency and inter-group divergence (Figure 7C). Differential expression analysis identified 2,446 upregulated and 2,327 downregulated genes in the CPB group compared to the sham group (Figures 7D–7F). Gene Ontology (GO) and Kyoto Encyclopedia of Genes and Genomes (KEGG) functional enrichment analyses mapped these differentially expressed genes (DEGs) to multiple cellular and metabolic processes (Figures 7G and 7H).

**Figure 7:**
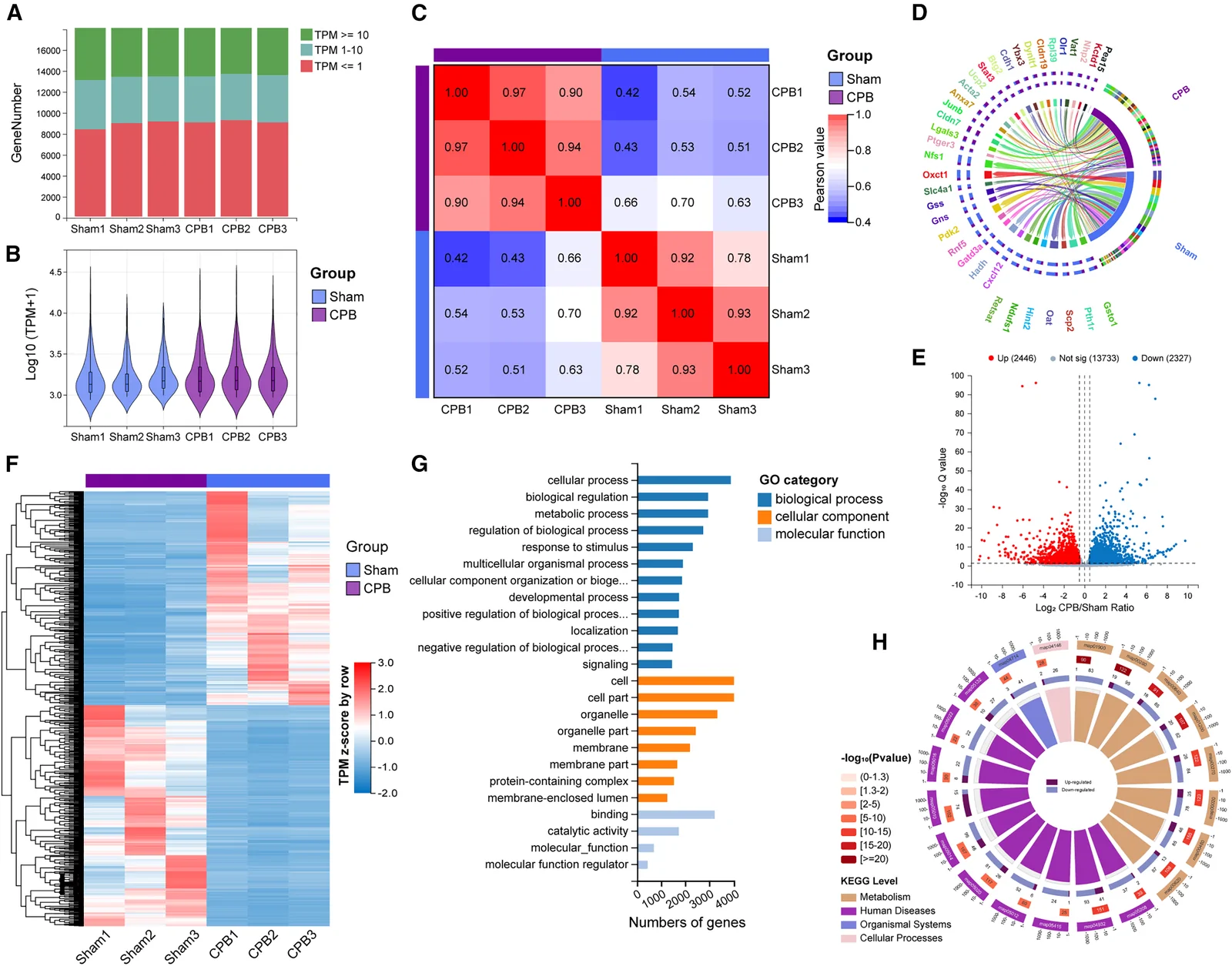
Transcriptomic profiling reveals distinct genome-wide expression signatures in CPB-induced AKI (A and B) Global gene expression distribution and quality assessment. (A) Stacked bar chart detailing the number of genes categorized by transcript abundance thresholds (transcripts per million [TPM] values). (B) Violin plots illustrating the density distribution of log10(TPM+1)-normalized expression levels across all biological replicates. (C) Pearson correlation matrix demonstrating robust intra-group reproducibility and significant inter-group transcriptomic divergence. (D) Chord diagram highlighting the relative expression patterns and group associations of top representative DEGs. (E) Volcano plot visualizing the global landscape of DEGs. Red and blue dots denote significantly upregulated (2,446) and downregulated (2,327) genes, respectively. (F) Hierarchical clustering heatmap of all identified DEGs, revealing complete transcriptomic segregation between the CPB and sham cohorts. (G) GO functional enrichment analysis categorizing DEGs into biological processes, cellular components, and molecular functions. (H) Circular visualization of KEGG pathway enrichment analysis, delineating the principal altered biological and metabolic networks. All transcriptomic data are derived from *n =* 3 independent biological replicates per group.

### Transcriptomic profiling reveals glycolytic reprogramming and the activation of inflammatory responses in CPB-AKI

Pathway analyses indicated that CPB-AKI is characterized by glycolytic reprogramming and enrichment of the inflammatory response pathway. Analysis of DEGs involved in energy metabolism showed an upregulation of genes associated with the pyruvate metabolism pathway (Figure 8A), the HIF-1 signaling pathway (Figure 8B), and the glycolysis/gluconeogenesis pathway (Figure 8C) in the CPB group. The intersection of the DEGs from these three metabolic pathways identified four overlapping genes: *Ldha*, *Ldhb*, *Pdhb*, and *Pdha1*. Among them, *Ldha* was significantly upregulated in the CPB group, whereas the other three genes were downregulated (Figure 8D).

**Figure 8:**
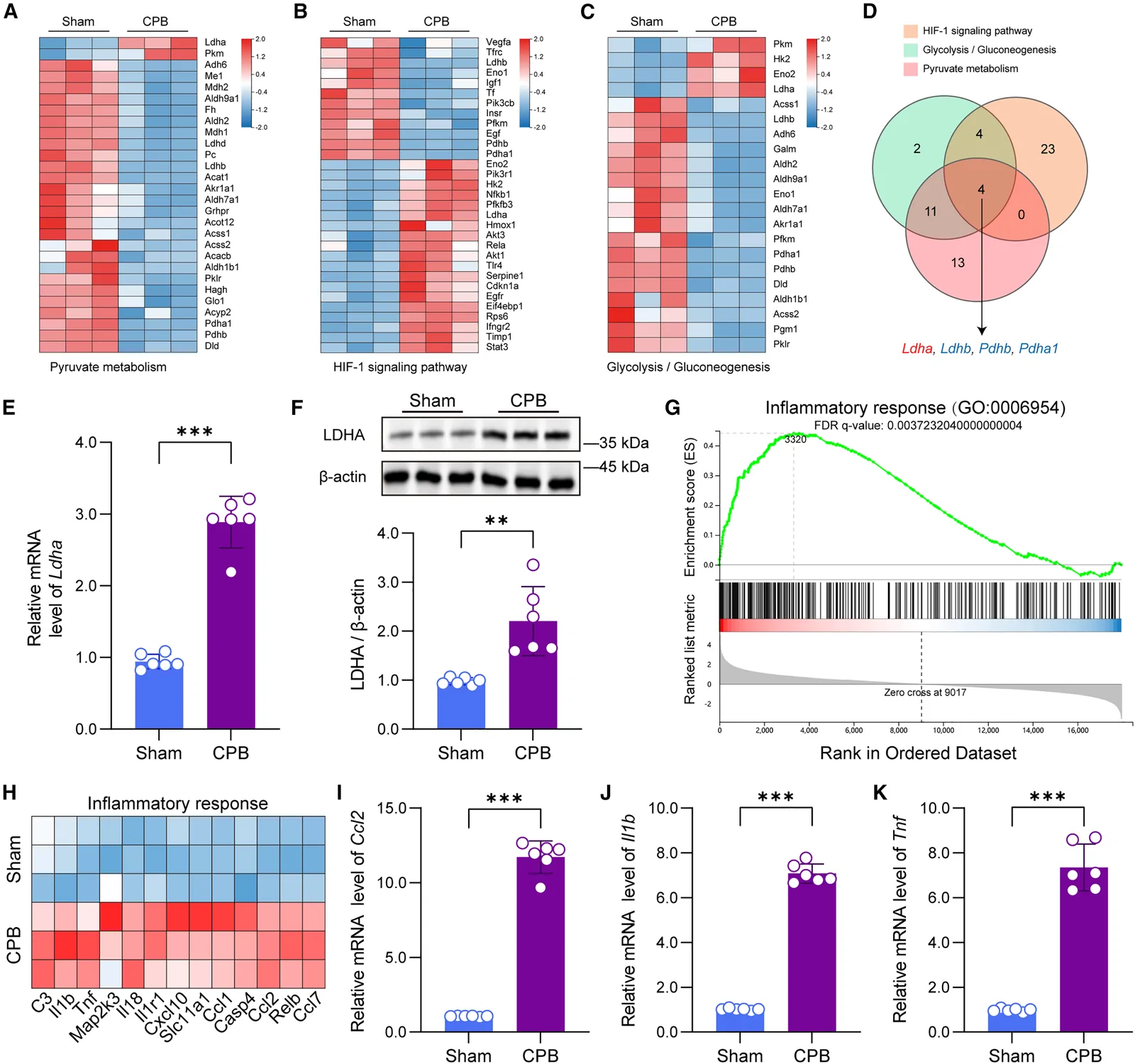
Transcriptomic profiling reveals glycolytic reprogramming and the activation of inflammatory responses in CPB-AKI (A–C) Heatmaps illustrating the expression profiles of DEGs significantly enriched in pyruvate metabolism (A), the HIF-1 signaling pathway (B), and glycolysis/gluconeogenesis (C). (D) Venn diagram identifying four core overlapping genes among these three key metabolic pathways. The gene symbol in red (*Ldha*) indicates marked upregulation, whereas those in blue (*Ldhb*, *Pdhb*, and *Pdha1*) indicate downregulation following CPB. (E) Relative mRNA expression of *Ldha* in renal tissues determined by RT-qPCR. *n =* 6; mean ± SD; unpaired *t* test. (F) Representative western blotting images and corresponding densitometric quantification of LDHA protein expression. *n =* 6; mean ± SD; unpaired *t* test. (G) GSEA plot demonstrating robust enrichment of the inflammatory response signature (GO:0006954) in the CPB group. (H) Heatmap depicting the relative expression levels of representative DEGs associated with the inflammatory response. (I–K) Relative mRNA expression levels of the pro-inflammatory chemokines and cytokines *Ccl2* (I), *Il1b* (J), and *Tnf* (K) in renal tissues. *n =* 6; mean ± SD; unpaired *t* test. ∗∗*p* < 0.01, ∗∗∗*p* < 0.001.

Based on the ML findings identifying lactate as a primary predictor of CPB-AKI, subsequent investigation focused on LDHA, an enzyme that catalyzes the conversion of pyruvate to lactate. Experimental validation confirmed that both mRNA and protein levels of LDHA were elevated in the renal tissues of CPB rats (Figures 8E and 8F). Concurrent with metabolic reprogramming, gene set enrichment analysis (GSEA) indicated the activation of the inflammatory response pathway following CPB (Figure 8G), supported by the clustering of inflammation-related genes (Figure 8H). *In vivo* analysis confirmed elevated mRNA levels of pro-inflammatory cytokines, including CCL2 (Figure 8I), IL-1β (Figure 8J), and TNF-α (Figure 8K), in the renal tissues of the CPB group. Collectively, these findings indicate that CPB induces LDHA-dependent glycolytic reprogramming and lactate overproduction, accompanied by a renal inflammatory response.

### Inhibition of lactate production mitigates renal injury and oxidative stress after CPB *in vivo*

Hexokinase 2 (HK2) and LDHA, two core glycolytic enzymes, are targeted and inhibited by 2-deoxy-D-glucose (2-DG) and OXA, respectively. Treatment with these two inhibitors effectively attenuates glycolytic flux and suppresses lactate generation. It has been shown that administration of 2-DG and OXA markedly lowers circulating lactate levels in inflammatory animal models and confers partial protection against organ damage.^18^^,^^19^ To evaluate whether lactate acts as a mechanistic mediator of CPB-AKI and to assess the experimental rigor of targeting lactate production, OXA or 2-DG was administered in the rat model (Figures 9A and 9B).

**Figure 9:**
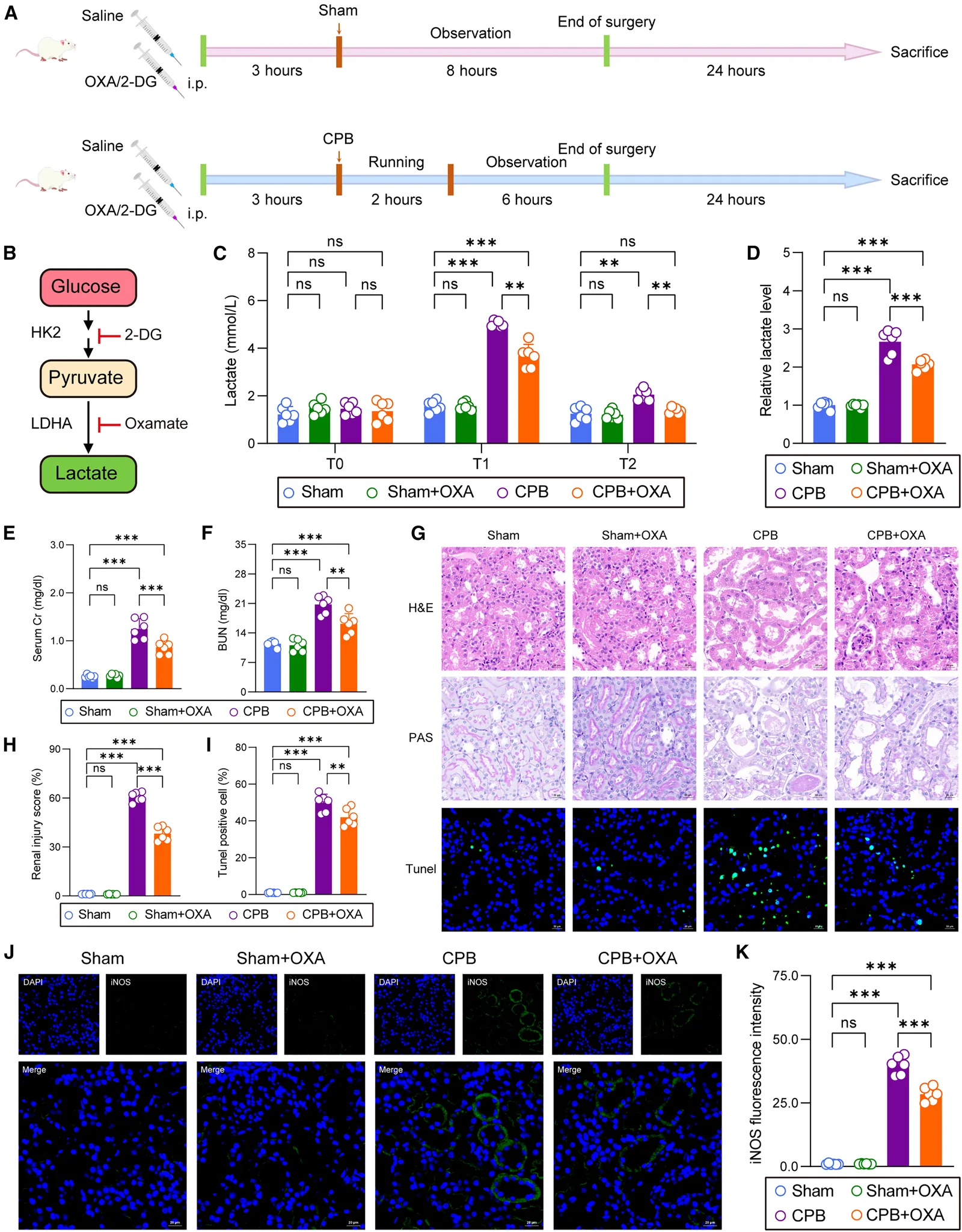
Inhibition of lactate production mitigates renal injury and oxidative stress after CPB *in vivo* (A) Schematic timeline of the *in vivo* pharmacological intervention (OXA or 2-DG) and experimental design. (B) Mechanistic schematic illustrating the targeted inhibition of lactate dehydrogenase A (LDHA)-mediated lactate production by OXA or 2-DG. (C) Dynamic trajectory of arterial blood lactate concentrations across the four experimental groups at three critical time points: 10 min after CPB initiation (T0), the end of CPB (T1), and 24 h post-surgery (T2). *n =* 6; mean ± SD; two-way ANOVA with Tukey’s test. (D) Relative lactate concentrations quantified in kidney tissues harvested at 24 h post-operation. *n =* 6; mean ± SD; one-way ANOVA with Tukey’s test. (E and F) Serum concentrations of Scr (E) and BUN (F) assessed at 24 h post-operation to evaluate renal function. *n =* 6; mean ± SD; one-way ANOVA with Tukey’s test. (G) Representative histological images of H&E staining, PAS staining, and TUNEL fluorescence in renal tissues. Scale bars, 20 μm. (H and I) Semi-quantitative analysis of the renal tubular injury score (H) and the percentage of TUNEL-positive apoptotic cells (I). *n =* 6; mean ± SD; one-way ANOVA with Tukey’s test. (J and K) Representative immunofluorescence images (J) and corresponding quantitative fluorescence intensity analysis (K) demonstrating the expression of the oxidative stress marker iNOS (green) in renal sections. Nuclei were counterstained with DAPI (blue). Scale bars, 20 μm. *n =* 6; mean ± SD; one-way ANOVA with Tukey’s test. ∗∗*p* < 0.01, ∗∗∗*p* < 0.001; ns, not significant. See also Figure S3.

Pre-treatment with OXA partially reduced the CPB-induced elevation in systemic blood lactate levels during and after the surgery (Figure 9C) and modestly decreased lactate accumulation within renal tissues at 24 h post-operation (Figure 9D). Consistent with this, administration of the second inhibitor, 2-DG, also achieved a comparable modest reduction in both systemic and local renal lactate levels (Figures S3A and S3B). This partial inhibition of lactate production was associated with renal structural and functional protection. The elevated levels of Scr and BUN induced by CPB were attenuated in the CPB+OXA group (Figures 9E and 9F). This functional protection was corroborated by the 2-DG intervention, which similarly reduced Scr and BUN levels (Figures S3C and S3D). Histological evaluations via H&E and PAS staining showed that both OXA (Figures 9G and 9H) and 2-DG (Figures S3E and S3F) ameliorated CPB-induced tubular damage. Furthermore, TUNEL staining indicated that OXA partially reduced CPB-induced renal cell apoptosis (Figures 9G and 9I).

To assess the impact on oxidative stress, the expression of inducible nitric oxide synthase (iNOS) was evaluated. Immunofluorescence staining demonstrated that the iNOS signals in the CPB group were partially reduced following OXA administration (Figures 9J and 9K). These results indicate that blocking lactate production mitigates CPB-induced structural kidney damage and oxidative stress.

### Inhibition of lactate production attenuates the inflammatory response and decreases global protein lactylation after CPB

We further investigated the effect of lactate inhibition on the specific biomarker profile and inflammatory response of CPB-AKI. Immunofluorescence staining showed a reduction in the expression of the AKI markers KIM-1 (Figure 10A) and NGAL (Figure 10B) in the renal tissues of the CPB+OXA group compared to the vehicle-treated CPB group. RT-qPCR analysis revealed that the upregulated mRNA levels of pro-inflammatory cytokines, including *Ccl2* (Figure 10C), *Il1b* (Figure 10D), and *Tnf* (Figure 10E), were decreased following OXA treatment. These data also demonstrated that OXA administration in sham-operated rats (sham+OXA group) did not alter baseline levels of Scr, BUN, or histological injury scores (Figures 9 and 10A–10E), indicating a favorable safety profile of this intervention under non-injured conditions. To evaluate the relationship between lactate accumulation and epigenetic modifications, the Pan-Kla status was assessed. Western blotting analysis showed a CPB-induced increase in Pan-Kla levels across the kidney tissue, which was attenuated in the CPB+OXA group (Figures 10F and 10G). Immunofluorescence staining localized this lactylation primarily to the renal tubular epithelial cells (Figures 10H and 10I). Both tissue-level and cellular-level lactylation signals were reduced by OXA pre-treatment. These *in vivo* findings indicate that lactate accumulation is associated with exacerbated renal injury, inflammation, and tubular lactylation.

**Figure 10:**
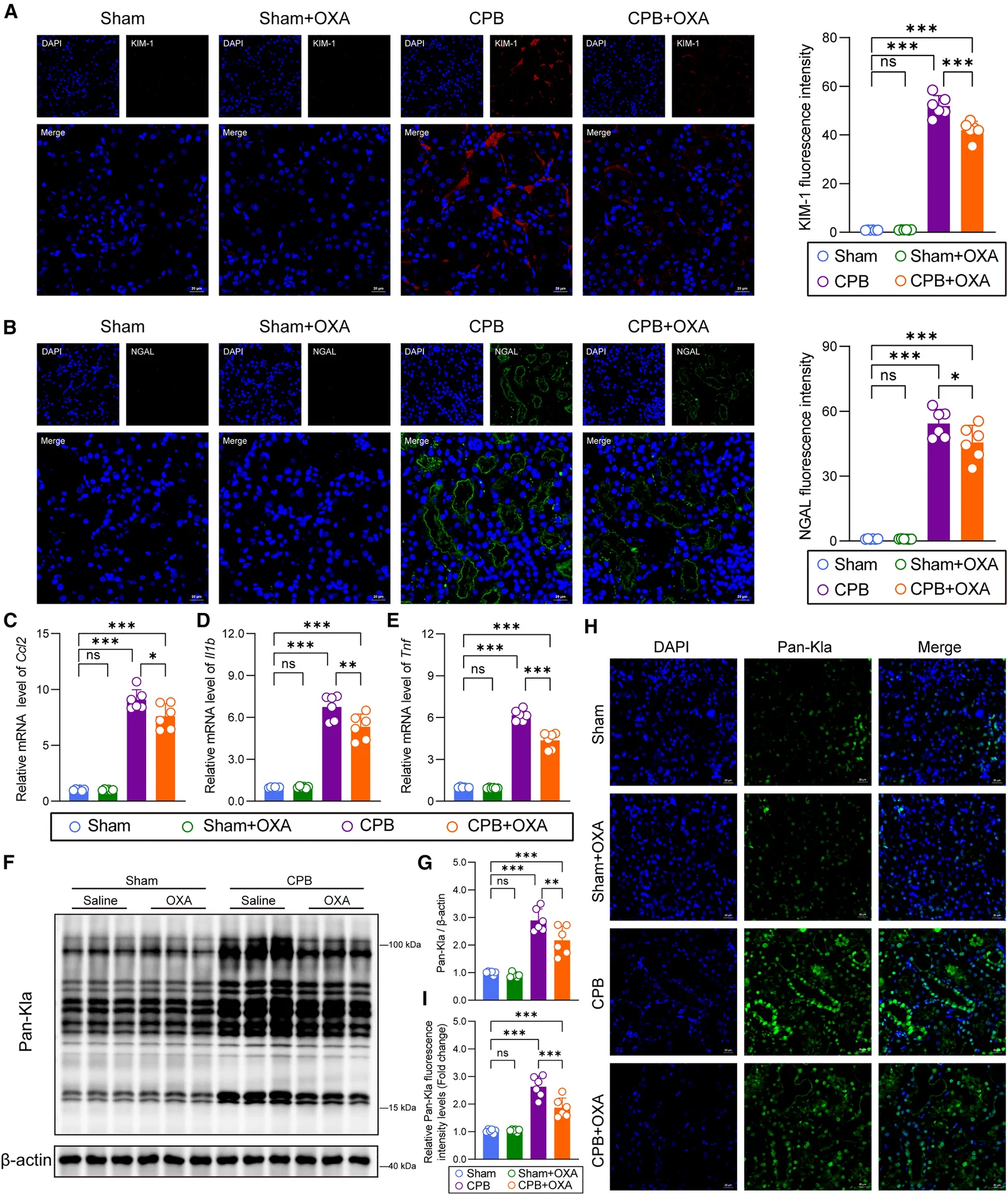
Inhibition of lactate production attenuates renal injury, inflammatory response, and decreases global protein lactylation after CPB (A and B) Representative confocal immunofluorescence images and corresponding quantitative fluorescence intensity analysis of the tubular injury markers KIM-1 (red; A) and NGAL (green; B) in renal sections. Nuclei were counterstained with DAPI (blue). Scale bars, 20 μm. *n =* 6; mean ± SD; one-way ANOVA with Tukey’s test. (C–E) Relative mRNA expression levels of the pro-inflammatory markers *Ccl2* (C), *Il1b* (D), and *Tnf* (E) in renal tissues, determined by RT-qPCR. *n =* 6; mean ± SD; one-way ANOVA with Tukey’s test. (F and G) Representative western blotting images (F) and corresponding densitometric quantification (G) of Pan-Kla levels in kidney tissues. *n =* 6; mean ± SD; one-way ANOVA with Tukey’s test. (H and I) Representative confocal immunofluorescence images (H) and quantitative fluorescence analysis (I) demonstrating the spatial distribution and expression intensity of Pan-Kla (green) specifically within renal tubular epithelial cells. Nuclei were counterstained with DAPI (blue). Scale bars, 20 μm. *n =* 6; mean ± SD; one-way ANOVA with Tukey’s test. ∗*p* < 0.05, ∗∗*p* < 0.01, ∗∗∗*p* < 0.001, ns, not significant.

### Inhibition of lactate production alleviates OGD/R-induced cellular injury, oxidative stress, and global protein lactylation *in vitro*

To exclude potential off-target effects of pharmacological inhibition and to investigate the role of the LDHA-driven lactate-lactylation axis, *in vitro* genetic silencing experiments were performed. HK-2 cells were subjected to OGD/R to mimic CPB-induced ischemic and hypoxic injury.^20^ The experimental flowchart, including transfection with LDHA-specific small interfering RNA (si-LDHA) or negative control RNA (si-NC), is presented in Figure 11A.

**Figure 11:**
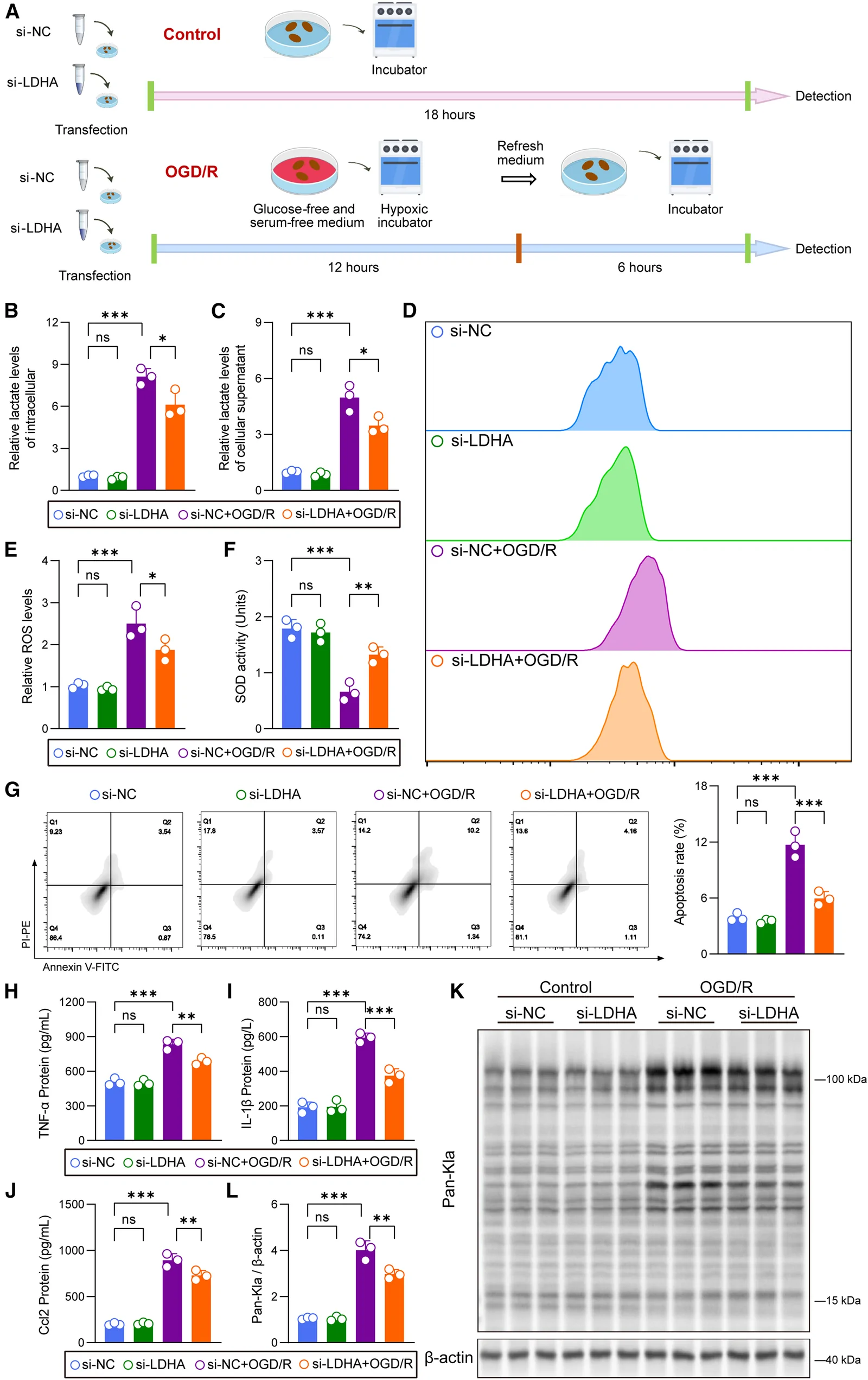
Inhibition of lactate production alleviates OGD/R-induced cellular injury, oxidative stress, and global protein lactylation *in vitro* (A) Schematic illustration of the *in vitro* experimental design involving targeted LDHA knockdown via small interfering RNA (siRNA) transfection and subsequent OGD/R treatment in HK-2 cells. (B and C) Relative intracellular (B) and extracellular (cell supernatant) (C) lactate concentrations across the four experimental groups. *n =* 3; mean ± SD; one-way ANOVA with Tukey’s test. (D and E) Representative flow cytometry histograms (D) and corresponding quantitative analysis (E) of intracellular ROS levels. *n =* 3; mean ± SD; one-way ANOVA with Tukey’s test. (F) SOD activity. *n =* 3; mean ± SD; one-way ANOVA with Tukey’s test. (G) Representative flow cytometry dot plots and quantitative analysis of apoptosis rates, assessed via annexin V-fluorescein isothiocyanate (FITC)/PI dual staining. *n =* 3; mean ± SD; one-way ANOVA with Tukey’s test. (H–J) Protein concentrations of the pro-inflammatory markers TNF-α (H), IL-1β (I), and CCL2 (J) in HK-2 cells, quantified by ELISA. *n =* 3; mean ± SD; one-way ANOVA with Tukey’s test. (K and L) Representative western blotting images (K) and densitometric quantification (L) of global pan-lysine lactylation (Pan-Kla) levels. *n =* 3; mean ± SD; one-way ANOVA with Tukey’s test. ∗*p* < 0.05, ∗∗*p* < 0.01, ∗∗∗*p* < 0.001; ns, not significant.

Consistent with the *in vivo* findings, OGD/R exposure resulted in an elevation of both intracellular and supernatant lactate levels in the si-NC+OGD/R group (Figures 11B and 11C). Silencing LDHA attenuated this lactate accumulation. We subsequently evaluated the effect of LDHA knockdown on cellular oxidative stress and apoptosis. Flow cytometry analysis using the CellROX probe demonstrated that OGD/R induced intracellular reactive oxygen species (ROS) generation (Figures 11D and 11E), accompanied by a reduction in superoxide dismutase (SOD) activity (Figure 11F). Transfection with si-LDHA partially reduced OGD/R-induced HK-2 cell apoptosis, as indicated by annexin V/phosphatidylinositol (PI) double staining (Figure 11G). Silencing LDHA in normoxic control cells (si-LDHA group) did not alter basal cell viability.

Furthermore, the *in vitro* inflammatory response triggered by OGD/R, characterized by elevated intracellular levels of TNF-α (Figure 11H), IL-1β (Figure 11I), and CCL2 (Figure 11J), was reduced upon LDHA silencing. To evaluate the epigenetic regulatory mechanism at the cellular level, global protein lactylation was assessed. Western blotting analysis showed that OGD/R treatment elicited a partial upregulation of Pan-Kla levels in HK-2 cells, which was attenuated in the si-LDHA+OGD/R group (Figures 11K and 11L). Collectively, these *in vitro* findings indicate an association between the lactate-lactylation axis and CPB-induced renal tubular injury and inflammation.

## Discussion

The pathogenesis of CPB-AKI remains complex, underscoring a clinical need for predictive tools and targeted interventions. The present study employed a “clinical-to-bench” translational approach to investigate the pathogenic role of lactate in CPB-AKI. We integrated multi-center intensive care databases (MIMIC-IV and MIMIC-III) with ML and SHAP algorithms, corroborated by transcriptomic profiling and *in vivo* and *in vitro* interventional models. This approach yielded three principal findings: first, ML models identified lactate as a top-ranking clinical predictor of CPB-AKI risk; second, mechanistic experiments confirmed that CPB induces LDHA-dependent glycolytic reprogramming, resulting in lactate accumulation within the renal microenvironment; and third, we demonstrated that this accumulated lactate is closely associated with renal inflammation and oxidative stress, accompanied by an elevation in global protein lactylation. Targeted inhibition of the LDHA-lactate-lactylation axis mitigated CPB-induced renal injury, offering a potential target for the prediction and management of this complication.

Early risk stratification is necessary for mitigating CPB-AKI and improving perioperative outcomes. However, current studies have predominantly focused on AKI following general cardiac surgery,^21^^,^^22^ with less emphasis on AKI specifically associated with CPB. Our study observed an incidence of CPB-AKI of 74.2%, which exceeds the generally reported rates of overall cardiac surgery-associated AKI. This prevalence highlights the limitations of traditional clinical scoring systems. In this context, our GBM model demonstrated high discrimination (testing AUC = 0.898) and clinical net benefit. Furthermore, SHAP analysis identified elevated preoperative lactate as a continuous predictor of CPB-AKI.

Recent literature applies ML to predict perioperative AKI using data-driven models^23^^,^^24^ that identify statistical associations without biological validation.^25^ Historically, elevated lactate in intensive care has often been interpreted as a surrogate biomarker of tissue hypoxia, hypoperfusion, or anaerobic metabolism.^26^^,^^27^^,^^28^ The translational logic of this study distinguishes lactate as a clinical risk predictor from its role as a mechanistic mediator. In the clinical analysis, elevated preoperative systemic lactate serves as a predictive biomarker for CPB-AKI risk. In the experimental models, local renal lactate accumulation post-CPB operates as a mechanistic mediator of tubular injury. This study combined clinical data analytics with *in vivo* and *in vitro* experiments to address whether lactate acts as a predictive biomarker or a mechanistic contributor to renal injury.

We investigated the origins and consequences of lactate accumulation in the CPB-injured kidney. The ischemic, hypoxic, and inflammatory conditions associated with CPB induce a metabolic shift toward aerobic glycolysis in renal tubular epithelial cells.^29^ Our transcriptomic analysis confirmed this glycolytic reprogramming and revealed an upregulated expression of the terminal glycolytic enzyme LDHA, which catalyzes lactate production, in the kidneys of the CPB group. Consequently, we targeted LDHA to modulate the intracellular lactate pool to determine whether lowering lactate levels could attenuate disease progression. However, it is crucial to note that the reduction in lactate levels following LDHA inhibition observed in our study should be interpreted as a targeted modulation of secondary metabolic reprogramming, rather than a reversal of the primary CPB-induced ischemic and hypoxic insult. In the context of CPB-AKI, lactate accumulation initially reflects upstream derangements, including hypoperfusion, hypoxia, and mitochondrial dysfunction. By lowering lactate levels (pharmacologically *in vivo* and genetically *in vitro*), we specifically intervened in the downstream metabolic cascade. This supports a re-evaluation of the role of lactate in the perioperative setting.^30^ Our *in vivo* and *in vitro* validations align with this framework, indicating that post-CPB lactate accumulation is closely associated with acute tubular necrosis and oxidative stress rather than a passive bystander.

It is imperative to recognize that the role of LDHA and lactate metabolism in AKI is highly complex and likely context dependent. A recent study demonstrated that the loss of proximal tubule LDHA actually exacerbates cisplatin-induced nephrotoxic AKI through metabolic dysregulation.^31^ The apparent discrepancy between their findings and the protective effects of LDHA inhibition observed in our CPB-AKI model can be attributed to several critical factors. First, this discrepancy stems from differing injury models and interventions. CPB-AKI involves acute ischemia and systemic inflammation, whereas cisplatin causes direct nephrotoxicity and DNA damage. Furthermore, our approach utilized acute, partial LDHA inhibition during an excessive lactate surge to blunt a maladaptive inflammatory loop. The systemic administration of OXA and 2-DG reduces systemic lactate exposure and local renal lactate production. The *in vivo* design does not differentiate the contribution of systemic exposure reduction from local production reduction. The *in vitro* HK-2 cell experiments demonstrate local lactate reduction. Clarifying the precise *in vivo* mechanism requires kidney-targeted lentiviral LDHA knockdown or kidney-specific LDHA knockout models. In contrast, complete genetic deletion abolishes baseline LDHA activity, which is essential for preserving glycolytic flux, NAD+/NADH redox balance, and overall metabolic flexibility. Thus, while hyper-lactylation amplifies inflammatory injury in CPB-AKI, basal lactate metabolism remains vital for tubular stress adaptation. Consequently, LDHA inhibition is not universally protective and demands highly precise timing and titration. Consistent with this partial inhibition, the decrease in lactate levels is modest. The downstream effect on markers of renal injury is modest. The observation of this protective effect across multiple measures of renal injury supports the conclusion that the effect is biological and not an artifact.

The mechanistic link between intracellular lactate accumulation and CPB-induced renal damage involves epigenetic regulation. Recent studies have identified lactate as a structural precursor for protein lactylation, a post-translational modification that modulates gene transcription and immune responses.^32^^,^^33^ Emerging literature links elevated lactylation to the exacerbation of inflammatory responses and macrophage activation in acute organ injuries, contributing to a pro-inflammatory response.^34^^,^^35^ Consistent with these findings, our study observed an increase in global protein lactylation within CPB-injured kidneys, which strongly correlated with oxidative stress and the release of pro-inflammatory cytokines. Reduction of lactate levels partially suppressed global lactylation, which was associated with attenuated cellular damage.

In conclusion, our interpretable ML models identify lactate as a top clinical predictor of CPB-AKI. Mechanistically, the experimental data indicate an association among lactate accumulation, protein lactylation, and renal injury. The lactate-lactylation axis represents a potential mechanistic pathway and candidate therapeutic target that requires further validation.

### Limitations of the study

Certain limitations must be acknowledged. First, the retrospective nature of the MIMIC databases may introduce confounding biases; future prospective, multicenter cohorts are required to validate the predictive threshold of lactate. Second, the MIMIC databases are intensive care unit (ICU) focused and lack detailed intraoperative data. Key anesthesia and CPB parameters (such as precise CPB duration, aortic cross-clamp time, and the magnitude of intraoperative mean arterial pressure fluctuations) were unavailable; future inclusion of these variables will improve prediction. Third, potential systemic off-target effects of OXA and 2-DG remain, despite corroboration from *in vitro* si-LDHA experiments. We did not assess whether metabolic interventions influenced upstream determinants of ischemic injury. Studies employing conditional kidney-specific knockout models and physiological monitoring are required to isolate the *in vivo* role and elucidate the metabolic landscape. Finally, although our data demonstrate an association between elevated global protein lactylation and CPB-AKI, they do not confirm a direct causal relationship. Future experiments incorporating exogenous lactate rescue, specific histone mark profiling, and mass spectrometry-based substrate identification are required to delineate the downstream mechanisms.

## Resource availability

### Lead contact

Requests for further information and resources should be directed to and will be fulfilled by the lead contact, Guyan Wang (guyanwang2006@163.com).

### Materials availability

This study did not generate new, unique reagents.

### Data and code availability

• The MIMIC-III and MIMIC-IV datasets are publicly available from PhysioNet (https://physionet.org/), subject to data use agreements. The bulk RNA sequencing data have been submitted and are publicly available via the Gene Expression Omnibus (GEO) GSE338239.
• This paper does not report original code.
• Any additional information required to reanalyze the data reported in this paper is available from the lead contact upon request.

## Acknowledgments

This study was supported by grants from the 10.13039/501100001809National Natural Science Foundation of China (grant nos. 82570468 and 82270411). The authors thank the developers and maintainers of the MIMIC-III and MIMIC-IV databases for creating and sharing these invaluable open-access critical care resources.

## Author contributions

Conceptualization, D.X., B.J., and G.W.; data curation, D.X.; formal analysis, D.X. and X.C.; methodology, D.X., M.Z., and T.W.; software, D.X., X.C., and T.W.; validation, D.X., X.C., and T.W.; investigation, M.Z. and G.W.; visualization, M.Z. and B.J.; resources, B.J. and G.W.; project administration, G.W.; supervision, G.W.; writing – original draft, D.X.; writing – review and editing, B.J. and G.W.

## Declaration of interests

The authors declare no competing interests.

## Declaration of generative AI and AI-assisted technologies in the writing process

During the preparation of this work, the authors used Gemini 3.1 Pro in order to check and refine the English text. After using this tool, the authors reviewed and edited the content as needed and take full responsibility for the content of the publication.

## STAR★Methods

### Key resources table

**Table:** 

REAGENT or RESOURCE	SOURCE	IDENTIFIER
**Antibodies**		

Rabbit anti-KIM-1	Abmart	Cat#PW12674; RRID: AB_3756731
Rabbit anti-NGAL	ImmunoWay Biotechnology	Cat#YM9036; RRID: AB_3751545
Rabbit anti-iNOS	Proteintech	Cat#18985-1-AP; RRID: AB_2782960
Rabbit anti-pan-lysine lactylation (Pan-Kla)	PTM Bio	Cat#1401RM; RRID: AB_2942013
Rabbit anti-LDHA	Proteintech	Cat#19987-1-AP; RRID: AB_10646429
Rabbit anti-β-actin	Proteintech	Cat#20536-1-AP; RRID: AB_10700003

**Chemicals, peptides, and recombinant proteins**		

Oxamate	Selleck Chemicals	Cat#S6871
2-DG	Selleck Chemicals	Cat#S4701
jetPRIME transfection reagent	Polyplus-transfection	Cat#101000046

**Critical commercial assays**		

Lactate Assay Kit	Nanjing Jiancheng Bioengineering	Cat#A019-2-1
SOD Assay Kit	Beyotime Biotechnology	Cat#S0101S
TRIzol Reagent	Thermo Fisher Scientific	Cat#15596018
Annexin V-FITC/PI Apoptosis Detection Kit	Solarbio	Cat#CA1020
CellROX Orange Flow Cytometry Assay Kit	Thermo Fisher Scientific	Cat#C10493
CCL2 ELISA kits	Proteintech	Cat#KE00425
IL-1β ELISA kits	Proteintech	Cat#KE00021
TNF-α ELISA kits	Proteintech	Cat#KE00154
*In situ* Cell Death Detection Kit (TUNEL)	Roche Diagnostics	Cat#11684817910

**Deposited data**		

MIMIC-IV critical care database	PhysioNet	https://physionet.org/content/mimiciv/
MIMIC-III critical care database	PhysioNet	https://physionet.org/content/mimiciii/
RNA-seq	Gene Expression Omnibus	GSE338239

**Experimental models: Cell lines**		

Human renal proximal tubular epithelial cells (HK-2)	Procell	Cat#CL-0109

**Experimental models: Organisms/strains**		

Rat: Sprague-Dawley (SD)	Beijing Huafukang Laboratory Animal Corp.	Cat#22001A

**Oligonucleotides**		

Primer sequences for qRT-PCR primer	Table S4	N/A

**Software and algorithms**		

R (v4.5.2)	R Foundation for Statistical Computing	https://www.r-project.org/
PostgreSQL/SQL	PostgreSQL Global Development Group	https://www.postgresql.org/
GraphPad Prism	GraphPad Software	https://www.graphpad.com/
ImageJ	NIH	https://imagej.nih.gov/ij/
Illustrator	Adobe	https://www.adobe.com

### Experimental model and study participant details

The clinical data used for model development and internal validation were extracted from the MIMIC-IV (version 2.2) database.^36^^,^^37^ This database includes 431,231 hospital admissions for 299,712 patients at Beth Israel Deaconess Medical Center between 2008 and 2019. For external validation, we used an independent cohort from the MIMIC-III database,^38^ which included 26,836 admissions for 23,692 patients from 2001 to 2008. To ensure the independence of the cohort, we strictly excluded patients in MIMIC-III who overlapped with those in MIMIC-IV. The author (Dinghao Xue) completed the Collaborative Institutional Training Initiative (CITI) program (Record ID: 13941513) to obtain data access. The use of the MIMIC-IV and MIMIC-III databases was approved by the Institutional Review Boards of the Massachusetts Institute of Technology and Beth Israel Deaconess Medical Center, and informed consent was waived because all data were de-identified. Ethical approval for the secondary use of these databases in the present study was also waived by the Ethics Committee of Beijing Tongren Hospital (No. TREC2025-KY205).

#### Study population

This study included adult patients (age ≥18 years) who underwent cardiovascular surgery under CPB. The included surgical procedures were not restricted to a specific kind of disease but encompassed a diverse spectrum of conditions requiring such intervention, including coronary artery disease, valvular heart disease, aortic dissection, and ventricular septal defects. For patients with multiple ICU admissions, only the data from their first ICU admission were included in the analysis to prevent data duplication. The exclusion criteria were summarized as follows: (1) surgeries performed without CPB; (2) pre-existing AKI or CKD prior to surgery; (3) a postoperative ICU length of stay of ≤48 h; and (4) death within 48 h following surgery. The detailed patient screening and selection workflow for both datasets is illustrated in Figure 1.

#### CPB model

All animal care protocols and experimental procedures were strictly approved by the Ethics Committee for Animal Experiments of Beijing Tongren Hospital (Approval No. TRLAWEC2022-S123) and conformed to the relevant national and institutional regulatory standards for the care and use of laboratory animals. Adult male Sprague-Dawley (SD) rats (350–450 g, 12–14 weeks of age) were housed under standardized specific pathogen-free conditions and provided *ad libitum* access to standard rodent chow and tap water.

The rat CPB model was established with rigorous hemodynamic monitoring. Rats were anesthetized with 1.5% sevoflurane and endotracheally intubated using a 16-gauge catheter. Mechanical ventilation was maintained via an animal respirator with a tidal volume of 8 mL/kg, a positive end-expiratory pressure (PEEP) of 5 cmH_2_O, and a respiratory rate of 60 breaths/min. Core body temperature was continuously maintained at approximately 36°C using a heating pad. The left femoral artery was cannulated with a 24-gauge catheter for real-time arterial blood pressure monitoring. The extracorporeal circuit comprised a custom-made multi-hole venous catheter, a venous reservoir, a miniature rat membrane oxygenator, and a peristaltic roller pump. The circuit was primed with a standardized mixture consisting of 15 mL of 6% hydroxyethyl starch, 1 mL of heparin (250 IU/kg), and 5% sodium bicarbonate. For the CPB routing, blood was drained by gravity from the right atrium via the right external jugular vein into the reservoir. Oxygenated blood was subsequently infused back into the animal via a 20-gauge catheter inserted into the tail artery, maintained at a steady flow rate of 120–140 mL/kg/min for 2 h. Following the termination of CPB, the animals were continuously observed under 1.0% sevoflurane anesthesia for 6 h prior to being returned to their cages. In the Sham group, rats underwent identical anesthesia, mechanical ventilation, and surgical cannulation, but without the initiation of the extracorporeal circulation. Arterial blood samples were prospectively collected at three distinct time points: 10 min after CPB initiation (T0), at the exact conclusion of CPB (T1), and 24 h postoperatively (T2). At 24 h post-surgery, all animals were euthanized via decapitation to harvest blood and kidney tissue samples for subsequent analyses. The actual CPB rat model was shown in Figure S2.

#### *In vivo* pharmacological intervention

To investigate the pathogenic role of lactate in CPB-AKI, OXA (750 mg/kg) or 2-DG (500 mg/kg) was administered via intraperitoneal injection 3 h prior to the initiation of CPB to inhibit endogenous lactate production.^18^^,^^19^ Animals in the Sham group received an intraperitoneal injection of an equivalent volume of sterile normal saline.

#### Cell culture

The HK-2 cell line used in this study was listed in the key resources table. The HK-2 cells were cultured in Dulbecco’s Modified Eagle Medium (DMEM) supplemented with 10% FBS and 100 U/mL penicillin and 100 μg/mL streptomycin at 37°C in a 5% CO_2_ atmosphere. All cell lines used in this study were confirmed to be free of mycoplasma.

#### Transfection and OGD/R model

For targeted genetic intervention, HK-2 cells were transiently transfected with a specific si-LDHA prior to the initiation of the OGD/R challenge. The si-LDHA oligonucleotide (sense sequence: 5′-CAACUGCUGUCACCUUCUATT-3’; GenePharma, China) was utilized. Transfection was performed using the transfection reagent at a final working concentration of 50 nM, in strict accordance with the manufacturer’s standardized protocol.

To simulate CPB-induced ischemic and hypoxic stress *in vitro*, an OGD/R model was established. Briefly, HK-2 cells were washed, and the standard culture medium was replaced with glucose-free and serum-free medium to induce nutrient deprivation. The cells were then transferred to a hypoxic chamber continuously flushed with a gas mixture of 1% O_2_, 5% CO_2_, and 94% N_2_ for a 12-h OGD phase. Following this phase, the medium was replaced with complete DMEM, and the cells were returned to normoxic conditions (21% O_2_, 5% CO_2_) for a 6-h reoxygenation phase.

### Method details

#### Data extraction and cleaning

Structured Query Language (SQL) in PostgreSQL was utilized to meticulously extract comprehensive clinical data from both the MIMIC-IV and MIMIC-III databases. All candidate predictor variables used for model construction were strictly extracted from before the start of the outcome assessment window. Specifically, preoperative laboratory indicators, blood gas indicators, and clinical characteristics were all taken from the most recent available measurement within 24 h before the start of CPB. The variables extracted in this study were: (1) Demographic information: Age, gender, BMI, smoking history, and drinking history. (2) Comorbidities: Hypertension, diabetes mellitus, CHD, heart failure, cerebrovascular accident (CVA), and chronic obstructive pulmonary disease (COPD). (3) Preoperative Clinical Severity Scores: Sequential Organ Failure Assessment (SOFA) score and SAPS II. (4) Surgery type: CABG, valve surgery, aortic surgery, combined surgery, and others. (5) Preoperative Laboratory and Blood Gas Parameters: Lactate, hemoglobin, red blood cell (RBC) count, white blood cell (WBC) count, platelet count, Scr, BUN, glucose, sodium, potassium, chloride, international normalized ratio (INR), prothrombin time (PT), partial pressure of carbon dioxide (PaCO_2_), and partial pressure of oxygen (PaO_2_). For variables with a missing data rate below 20%, missing values were imputed using the Multiple Imputation by Chained Equations (MICE) framework via the predictive mean matching (PMM) method. A total of 5 imputed datasets (m = 5) with a maximum of 5 iterations were generated to mitigate bias while preserving statistical power.

#### Primary outcome definition

The primary clinical outcome of this study was the development of AKI following CPB surgery. The diagnosis of CPB-AKI was ascertained strictly according to the Kidney Disease: Improving Global Outcomes (KDIGO) clinical practice guidelines.^39^ Specifically, CPB-AKI was defined entirely based on perioperative fluctuations in Scr levels. A patient was diagnosed with AKI if their postoperative Scr level surged to ≥1.5 times the preoperative baseline level, or if an absolute increase in Scr of ≥0.3 mg/dL occurred within 48 h postoperatively. We did not use urine output criteria for AKI.

#### Feature selection

We employed a dual-feature selection strategy combining the Boruta algorithm and LASSO regression. First, the Boruta algorithm was applied to evaluate comprehensive feature importance by iteratively comparing the Z-scores of actual candidate variables against those of randomized “shadow features,” retaining only the variables that significantly outperformed their shadow counterparts. Concurrently, LASSO regression with 10-fold cross-validation was utilized to eliminate multicollinearity and extract potential linear predictors with non-zero coefficients. Ultimately, the intersecting variables independently identified by both methods were selected for the final predictive model construction.

#### Model construction strategy and evaluation

The primary MIMIC-IV dataset was randomly partitioned into a training cohort (70%) and an internal validation cohort (30%). Class imbalance within the training set was rigorously addressed using the Synthetic Minority Over-sampling Technique (SMOTE). Subsequently, eight supervised ML algorithms—GBM, LightGBM, Logistic Regression, CatBoost, SVM, XGBoost, Neural Network, and AdaBoost—were constructed. Model training incorporated a 10-fold cross-validation strategy coupled with grid search hyperparameter tuning, as detailed in Table S3, optimizing strictly for the maximum AUC to minimize evaluation bias and ensure generalizability. The optimized models, trained exclusively on the training set, were then comprehensively evaluated across both the internal and external (MIMIC-III) validation datasets. Predictive discrimination was quantified using the AUC, supplemented by a holistic suite of performance metrics including accuracy, sensitivity, specificity, precision, F1 score, and Cohen’s Kappa value. Furthermore, the true clinical net benefit and prognostic reliability of the best-performing model were assessed through DCA and calibration curves.

The SHAP method was used to visualize important individual features and quantify the contribution of each feature to the model’s predictions.

#### Renal function assessment

Blood samples were centrifuged at 3000 rpm for 10 min to isolate serum. Scr and BUN levels were subsequently quantified using an automated chemistry analyzer in strict accordance with the manufacturer’s protocols.

#### Lactate quantification and blood gas analysis

Arterial blood lactate concentrations were measured following sample acquisition using an i-STAT portable clinical analyzer equipped with CG4+ cartridges.

For the quantification of tissue and *in vitro* lactate levels, kidney tissues and harvested HK-2 cells were homogenized in lysis buffer and subjected to ultrasonic disruption on ice (300 W; 3 s on/7 s off cycles for 3 min). Following centrifugation at 12000 × g for 10 min at 4°C, the clarified lysates were collected. The lactate concentrations within these intracellular lysates and the cell culture supernatants were determined using a lactate assay kit according to the manufacturer’s instructions.

#### H&E staining and PAS staining

At 24 h post-CPB, kidney tissues from euthanized rats were fixed in 4% paraformaldehyde, embedded in paraffin, and sectioned at a thickness of 4 μm. For morphological evaluation, tissue sections were deparaffinized in xylene and rehydrated through a graded series of ethanol solutions before H&E and PAS staining.

Histopathological changes in the renal cortex and outer medulla were observed under a light microscope. The severity of renal tubular injury was semi-quantitatively assessed by two independent histologists blinded to the experimental group assignments. A scoring system was employed to quantify the percentage of damaged tubules (characterized by tubular dilation, necrosis, cast formation, and loss of brush border) in 10 randomly selected, non-overlapping fields per section. The injury score was graded on a scale of 0–4 based on the following criteria: Level 0, no damage; Level 1, <25% damage; Level 2, 25–49% damage; Level 3, 50–75% damage; and Level 4, >75% damage.

#### Immunofluorescence staining

Sections were deparaffinized in xylene, rehydrated through a graded ethanol series, and rinsed in deionized water. Antigen retrieval was performed via heat-induced epitope retrieval in a microwave oven using citrate buffer (pH 6.0). Non-specific binding sites were blocked with 5% normal goat serum for 1 h at room temperature. The sections were incubated overnight at 4°C with the following primary antibodies: rabbit anti-KIM-1 (1:200), rabbit anti-NGAL (1:200), rabbit anti-iNOS (1:200), and rabbit anti-pan-lysine lactylation (1:100). Following primary antibody incubation and washing, sections were incubated with corresponding Alexa Fluor-conjugated secondary antibodies for 1 h at room temperature in the dark. Nuclei were counterstained with DAPI. Fluorescence images were captured using a laser-scanning confocal microscope.

Quantitative analysis of fluorescence intensity was performed using ImageJ software. A macro was utilized to batch split color channels and calculate the mean optical density across randomly selected high-power fields for each section. The investigators responsible for image acquisition and subsequent quantitative analysis were blinded to the experimental group assignments.

#### TUNEL staining

To evaluate cellular apoptosis, kidney sections were stained using the *In Situ* Cell Death Detection Kit according to the manufacturer’s instructions. Nuclei were counterstained with DAPI. Fluorescence images were acquired using a confocal microscope. The apoptotic index was determined by calculating the ratio of TUNEL-positive cells to total DAPI-positive nuclei across randomly selected high-power fields.

#### Western blotting

HK-2 cells and renal tissues were lysed in RIPA buffer and centrifuged at 12000 rpm for 10 min at 4°C. Supernatants were collected, and total protein concentrations were quantified by BCA method. Equal amounts of protein samples were mixed with 4× loading buffer, separated by SDS-PAGE, and electrotransferred onto PVDF membranes. The membranes were blocked with 5% non-fat milk in Tris-buffered saline containing Tween 20 (TBST) for 2 h at 37°C, followed by overnight incubation at 4°C with the following primary antibodies: rabbit anti-pan-lysine lactylation (1:1000), rabbit anti-LDHA (1:2000), and rabbit anti-β-actin (1:10000). Following washes with TBST, the membranes were incubated with the corresponding fluorophore-conjugated secondary antibodies for 1 h at room temperature. Target protein bands were scanned and visualized using the LI-COR Odyssey Infrared Imaging System. Band optical densities were analyzed using ImageJ software.

#### qRT-PCR

Total RNA was isolated from cultured HK-2 cells and renal tissues using TRIzol Reagent. Following quantification and purity assessment, RNA was reverse-transcribed into cDNA according to the manufacturer’s protocol. qRT-PCR was performed on a Real-Time PCR System. Relative mRNA expression levels were calculated using the 2^−ΔΔCt^ method, with target gene expression normalized to the endogenous control, β-actin. All specific primer sequences utilized in this study are detailed in Table S4.

#### RNA-seq

Total RNA obtained from renal tissues was used for RNA-seq analysis. RNA extraction was performed using Trizol reagent, followed by purification of poly(A) RNA. The purified RNA was used to construct cDNA libraries. All samples were sequenced on the DNBSEQ platform (BGI-Shenzhen, China).

For the bioinformatic workflow, raw sequencing reads were filtered to remove adapter sequences and low-quality bases. The clean reads were aligned to the rat reference genome. Gene expression levels were quantified, and differential expression analysis was conducted using the DESeq2 package in R. Genes with a false discovery rate (FDR) < 0.05 were considered differentially expressed. GO, KEGG, and GSEA were conducted to map altered biological and metabolic networks.

#### Flow cytometry for apoptosis analysis

Flow cytometry was performed using the Annexin V-FITC/PI Apoptosis Detection Kit. Harvested cells were washed with cold PBS and resuspended in 100 μL of 1× Binding Buffer. Annexin V-FITC (5 μL) was added to the cell suspension, followed by a 15-min incubation at room temperature in the dark. Following the addition of 5 μL of PI and an additional 400 μL of 1× Binding Buffer, the samples were analyzed using a flow cytometer.^40^

#### CellROX (FACS)

ROS levels were quantified using the CellROX Orange Flow Cytometry Assay Kit. Following treatments, HK-2 cells were incubated with CellROX Orange reagent (5 μM) at 37°C for 30 min, washed with PBS, and analyzed using a flow cytometer. Relative ROS levels were determined based on the mean fluorescence intensity.

#### SOD assay

SOD activity in the collected HK-2 cell supernatants was quantified using a SOD Assay Kit (S0101S; Beyotime Biotechnology, China) according to the manufacturer’s protocol.

#### Enzyme-linked immunosorbent assay (ELISA)

The concentrations of inflammatory cytokines, including CCL2, IL-1β, and TNF-α, in the cell lysates were quantified using ELISA kits according to the manufacturer’s instructions.

### Quantification and statistical analysis

Clinical Data and ML: Clinical characteristics for non-normally distributed continuous variables were presented as medians with interquartile ranges (IQRs), while categorical variables were expressed as frequencies and percentages. For model construction, categorical variables were dummy-coded as 1 (yes) and 0 (no). Differences between groups were analyzed using the Mann-Whitney U test or Kruskal-Wallis test for continuous variables, and the Chi-square test or Fisher’s exact test for categorical variables. Clinical statistical analyses were performed using R software version 4.5.2. The LASSO regression and Boruta feature selection algorithms were implemented using the glmnet and Boruta R packages, respectively. Hyperparameter tuning for the ML models was performed via a grid search approach. The kernelshap and shapviz R packages were utilized to calculate SHAP values to determine variable importance and model interpretability.

Basic Research Data: Data from basic experiments were presented as the mean ± SD. Differences between two independent groups were evaluated using unpaired two-tailed *t* test. For comparisons among multiple groups, a one-way or two-way analysis of variance (ANOVA) followed by Tukey’s post hoc test was performed. A two-way ANOVA with Tukey’s post hoc test was used to assess group differences across multiple time points. Statistical analyses for basic research were conducted using GraphPad Prism 9.5. All statistical tests were two-tailed, and a *p*-value <0.05 was considered statistically significant.
