# Supplementary material for: Lactate as a predictive biomarker and therapeutic target in cardiopulmonary bypass-associated acute kidney injury
Source: iScience. 2026 Oct 8;29(11):117643. doi: 10.1016/j.isci.2026.117643 (PMC13676736; doi:10.1016/j.isci.2026.117643)

iScience, Volume *29*

Supplemental information

Lactate as a predictive biomarker and therapeutic target in cardiopulmonary bypass-associated acute kidney injury

Dinghao Xue, Mingru Zhang, Xinlu Chang, Tianlong Wang, Bingyang Ji, Chunhua Xi, and Guyan Wang

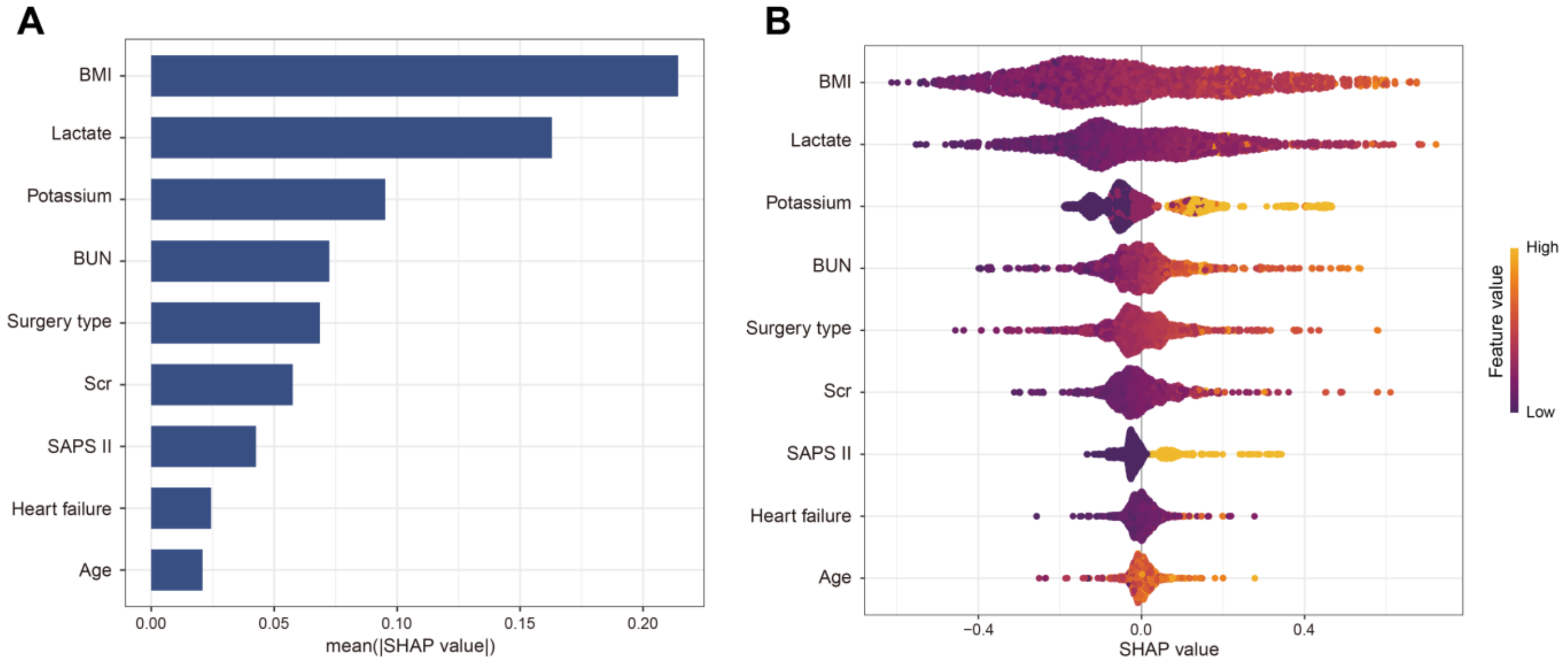

Figure S1. Global feature importance and interpretability analysis of the optimal GBM model via SHAP, related to Figure 4.

(A) Bar chart depicting the mean absolute SHAP values of the top nine predictors, ranked in descending order of their global contribution to the Gradient Boosting Machine (GBM) model.

(B) SHAP beeswarm summary plot illustrating the distribution of individual feature impacts across all patients. Each dot represents a single patient. The horizontal axis indicates the directional impact of a feature on the model's prediction of CPB-AKI risk, while the color gradient reflects the actual magnitude of the feature value (ranging from purple for low values to yellow for high values).

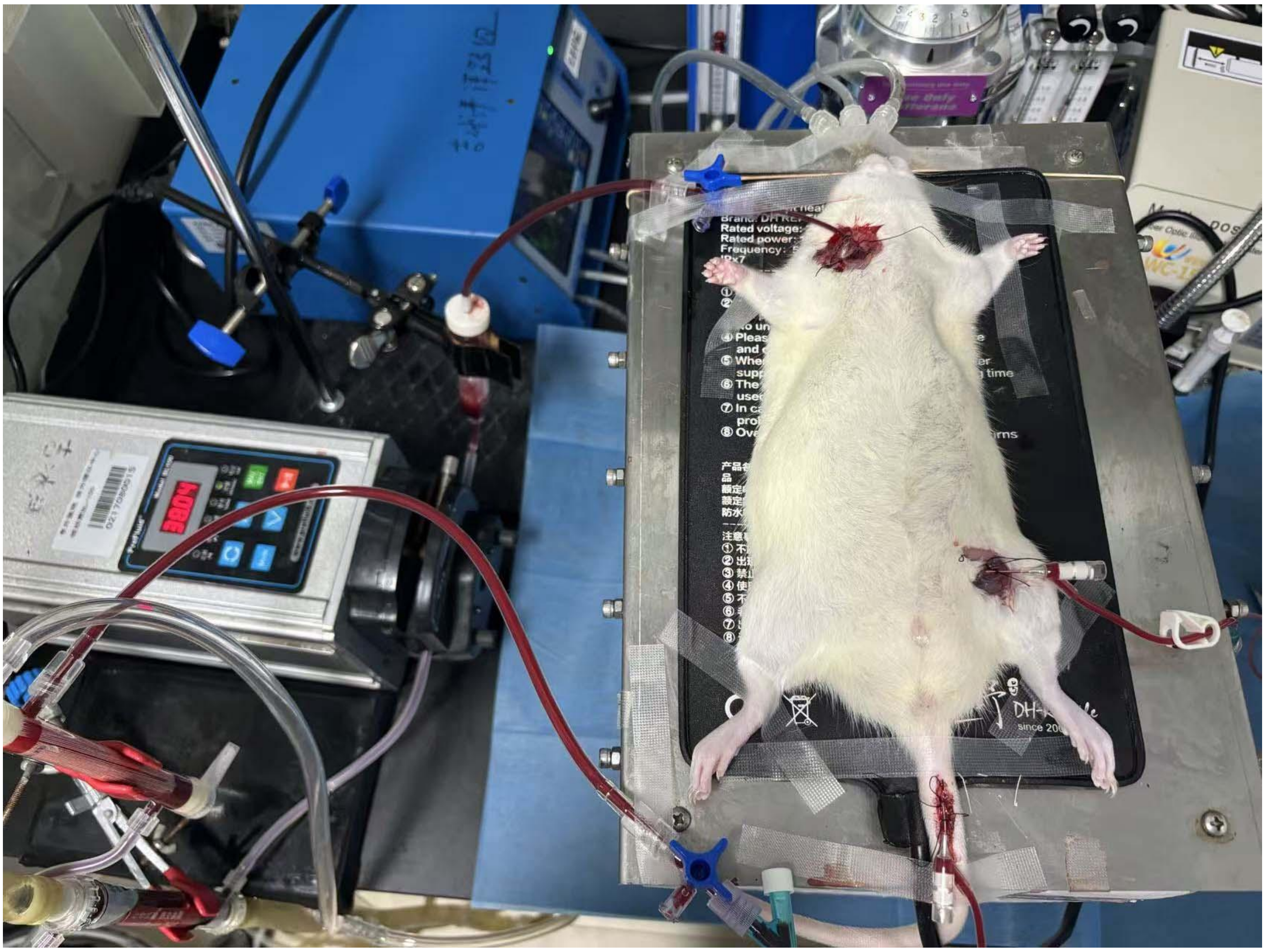

Figure S2. The whole CPB circuit, related to STAR Methods

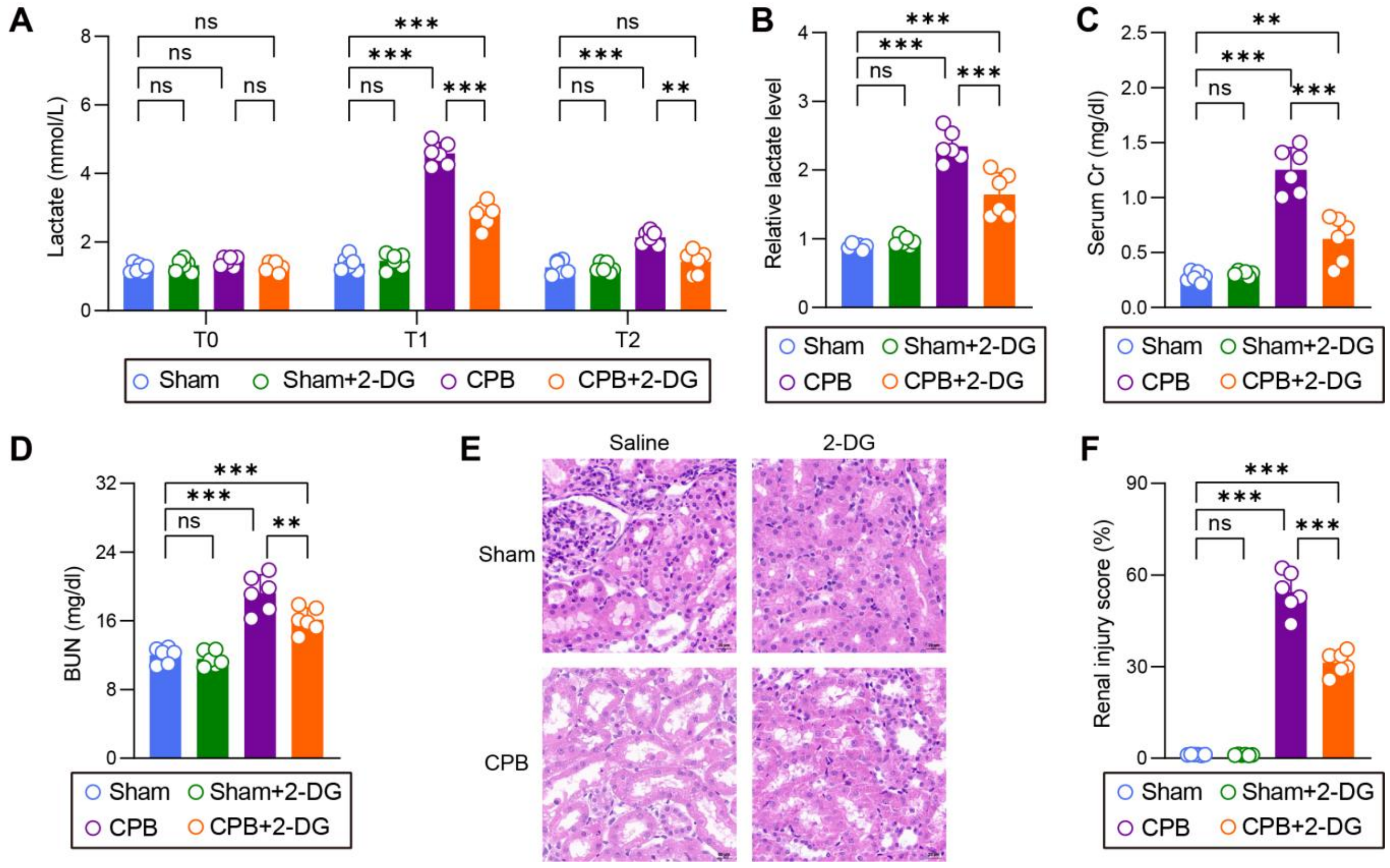

Figure S3. Inhibition of glycolysis by 2-DG mitigates CPB-induced lactate accumulation and renal injury *in vivo*, related to Figure 9.

(A) Dynamic trajectory of arterial blood lactate concentrations across the four experimental groups at three time points: 10 min after CPB initiation (T0), the end of CPB (T1), and 24 hours post-surgery (T2). $n = 6$, mean ± SD, two-way ANOVA with Tukey's test.

(B) Relative lactate concentrations quantified in kidney tissues harvested at 24 hours post-operation. $n = 6$, mean ± SD, one-way ANOVA with Tukey's test.

(C, D) Serum concentrations of Scr (C) and BUN (D) assessed at 24 hours post-operation. $n = 6$, mean ± SD, one-way ANOVA with Tukey's test.

(E) Representative histological images of H&E staining in renal tissues. Scale bars, 20 μm.

(F) Semi-quantitative analysis of the renal tubular injury score. $n = 6$, mean ± SD, one-way ANOVA with Tukey's test.

**P < 0.01, ***P < 0.001, ns, not significant.

Table S1 Baseline comparison of patients in MIMIC-IV and MIMIC-III datasets, related to Table 1.

Characteristics	Overall (n=6558)	MIMIC-IV (n=3613)	MIMIC-III (n=2945)	*P value*
Age, years (median [Q1,Q3])	69 (60-77)	68 (60-76)	69 (60-77)	0.897
Gender, n (%)				<0.001
Female	4431 (67.57)	2520 (69.75)	1911 (64.89)	
Male	2127 (32.43)	1093 (30.25)	1034 (35.11)	
BMI, kg/m² (median [Q1,Q3])	28.535 (25.306-32.485)	29.245 (25.970-33.145)	27.673 (24.547-31.555)	<0.001
Smoking history, n (%)				<0.001
No	4849 (73.94)	2368 (65.54)	2481 (84.24)	
Yes	1709 (26.06)	1245 (34.46)	464 (15.76)	
Drinking history, n (%)				0.359
No	6381 (97.30)	3509 (97.12)	2872 (97.52)	
Yes	177 (2.70)	104 (2.88)	73 (2.48)	
Hypertension, n (%)				0.274
No	2259 (34.45)	1266 (35.04)	993 (33.72)	
Yes	4299 (65.55)	2347 (64.96)	1952 (66.28)	
Diabetes mellitus, n (%)				0.147
No	4636 (70.69)	2527 (69.94)	2109 (71.61)	
Yes	1922 (29.31)	1086 (30.06)	836 (28.39)	
CHD, n (%)				<0.001
No	1645 (25.08)	987 (27.32)	658 (22.34)	
Yes	4913 (74.92)	2626 (72.68)	2287 (77.66)	
Heart failure, n (%)				<0.001
No	4982 (75.97)	2828 (78.27)	2154 (73.14)	
Yes	1576 (24.03)	785 (21.73)	791 (26.86)	
CVA, n (%)				<0.001
No	6126 (93.41)	3333 (92.25)	2793 (94.84)	
Yes	432 (6.59)	280 (7.75)	152 (5.16)	
COPD, n (%)				0.361
No	5992 (91.37)	3312 (91.67)	2680 (91.00)	
Yes	566 (8.63)	301 (8.33)	265 (9.00)	
SOFA score (median [Q1,Q3])	5 (3-7)	5 (3-7)	5 (3-7)	<0.001
SAPS II (median [Q1,Q3])	34 (28-42)	35 (28-42)	34 (28-42)	0.010
Lactate, mmol/L (median [Q1,Q3])	2.2 (1.5-2.9)	2.1 (1.4-2.8)	2.3 (1.6-3.0)	<0.001
Hemoglobin, g/dL (median [Q1,Q3])	11.2 (9.6-13.0)	11.3 (9.7-13.1)	11.1 (9.6-12.8)	0.001
Platelet, $\times 10^9$/L (median [Q1,Q3])	183 (141-232)	180 (140-226)	190 (143-240)	<0.001
RBC, $\times 10^{12}$/L (median [Q1,Q3])	3.72 (3.16-4.28)	3.75 (3.16-4.33)	3.69 (3.16-4.24)	0.002
WBC, $\times 10^9$/L (median [Q1,Q3])	9.6 (7.2-13.1)	9.4 (7.1-13.0)	9.7 (7.4-13.2)	0.013
Chloride, mmol/L (median [Q1,Q3])	106 (103-110)	107 (103-111)	106 (103-110)	<0.001
Glucose, mg/dL (median [Q1,Q3])	117 (100-139)	115 (100-134)	120 (100-145)	<0.001
Potassium, mmol/L (median [Q1,Q3])	4.2 (3.9-4.5)	4.1 (3.9-4.4)	4.2 (3.9-4.5)	<0.001
Sodium, mmol/L (median [Q1,Q3])	140 (138-141)	140 (138-141)	139 (137-141)	<0.001
$PaCO_2$, mmHg (median [Q1,Q3])	41 (37-45)	41 (38-45)	41 (37-45)	0.385
PaO_2, mmHg (median [Q1,Q3])	380 (307-430)	380 (311-429)	381 (301-433)	0.024
INR (median [Q1,Q3])	1.2 (1.1-1.4)	1.2 (1.1-1.4)	1.3 (1.1-1.5)	<0.001
PT, s (median [Q1,Q3])	14 (12.7-15.5)	13.8 (12.3-15.4)	14.2 (13.1-15.7)	<0.001
Scr, mg/dL (median [Q1,Q3])	0.9 (0.7-1.1)	0.9 (0.8-1.1)	0.9 (0.7-1.1)	<0.001
BUN, mg/dL (median [Q1,Q3])	17 (13-21)	17 (13-21)	17 (13-22)	0.005
Type of surgery, n (%)				<0.001
CABG	3270 (49.86)	1736 (48.05)	1534 (52.09)	
Valve surgery	1669 (25.45)	1072 (29.67)	597 (20.27)	
Aortic surgery	27 (0.41)	16 (0.44)	11 (0.37)	
Combined surgery	204 (3.11)	114 (3.16)	90 (3.06)	
Others	1388 (21.16)	675 (18.68)	713 (24.21)	
CPB-AKI, n (%)				<0.001
No	1173 (17.89)	932 (25.80)	241 (8.18)	
Yes	5385 (82.11)	2681 (74.20)	2704 (91.82)	

BMI, body mass index; *CHD*, coronary heart disease; *CVA*, cerebrovascular accident; *COPD*, chronic obstructive pulmonary disease; *SOFA*, Sequential Organ Failure Assessment; *SAPS II*, Simplified Acute Physiology Score II; *RBC*, red blood cell; *WBC*, white blood cell; $PaCO_2$, partial pressure of carbon dioxide in arterial blood; PaO_2, partial pressure of oxygen in arterial blood; *INR*, international normalized ratio; *PT*, prothrombin time; *Scr*, serum creatinine; *BUN*, blood urea nitrogen; *CABG*, coronary artery bypass grafting; *CPB-AKI*, cardiopulmonary bypass-associated acute kidney injury

Table S2 Baseline comparison of patients with and without CPB-AKI in MIMIC-IV dataset, related to Table 1.

Characteristics	Overall (n=3613)	CPB-AKI (n=2681)	Non-CPB-AKI (n=932)	*P value*
Age, years (median [Q1,Q3])	68 [60, 76]	69 [61, 77]	66 [58, 74]	<0.001
Gender, n (%)				0.009
Female	2520 (69.75)	1838 (68.56)	682 (73.18)	
Male	1093 (30.25)	843 (31.44)	250 (26.82)	
BMI, kg/m² (median [Q1,Q3])	29.245 [25.97, 33.145]	30.081 [26.563, 34.160]	27.252 [24.640, 30.311]	<0.001
Smoking history, n (%)				0.941
No	3509 (97.12)	2603 (97.09)	906 (97.21)	
Yes	104 (2.88)	78 (2.91)	26 (2.79)	
Drinking history, n (%)				0.074
No	2368 (65.54)	1780 (66.39)	588 (63.09)	
Yes	1245 (34.46)	901 (33.61)	344 (36.91)	
Hypertension, n (%)				0.384
No	1266 (35.04)	928 (34.61)	338 (36.27)	
Yes	2347 (64.96)	1753 (65.39)	594 (63.73)	
Diabetes mellitus, n (%)				0.339
No	3333 (92.25)	2466 (91.98)	867 (93.03)	
Yes	280 (7.75)	215 (8.02)	65 (6.97)	
CHD, n (%)				<0.001
No	2527 (69.94)	1830 (68.26)	697 (74.79)	
Yes	1086 (30.06)	851 (31.74)	235 (25.21)	
Heart failure, n (%)				<0.001
No	2828 (78.27)	2045 (76.28)	783 (84.01)	
Yes	785 (21.73)	636 (23.72)	149 (15.99)	
CVA, n (%)				0.615
No	987 (27.32)	726 (27.08)	261 (28.00)	
Yes	2626 (72.68)	1955 (72.92)	671 (72.00)	
COPD, n (%)				0.052
No	3312 (91.67)	2443 (91.12)	869 (93.24)	
Yes	301 (8.33)	238 (8.88)	63 (6.76)	
SOFA score (median [Q1,Q3])	5 [3, 7]	5 [4, 7]	4 [3, 6]	<0.001
SAPS II (median [Q1,Q3])	35 [28, 42]	36 [29, 44]	32 [26, 39]	<0.001
Lactate, mmol/L (median [Q1,Q3])	2.1 [1.6, 2.8]	2.1 [1.6, 2.8]	2.0 [1.6, 2.7]	<0.001
Hemoglobin, g/dL (median [Q1,Q3])	11.3 [9.7, 13.1]	11.3 [9.7, 13.0]	11.5 [9.7, 13.4]	0.098
Platelet, $\times 10^9$/L (median [Q1,Q3])	180 [140, 226]	181 [141, 227]	175 [139, 223]	0.946
RBC, $\times 10^{12}$/L (median [Q1,Q3])	3.75 [3.16, 4.33]	3.74 [3.17, 4.31]	3.75 [3.128, 4.36]	0.644
WBC, $\times 10^9$/L (median [Q1,Q3])	9.4 [7.1, 13.0]	9.5 [7.1, 13.1]	9.3 [7.075, 12.825]	0.129
Chloride, mmol/L (median [Q1,Q3])	107 [103, 111]	107 [103, 110]	107 [103, 111]	0.108
Glucose, mg/dL (median [Q1,Q3])	115 [100, 134]	116 [101, 135]	113 [100, 131]	0.007
Potassium, mmol/L (median [Q1,Q3])	4.1 [3.9, 4.4]	4.1 [3.9, 4.4]	4.1 [3.9, 4.4]	0.129
Sodium, mmol/L (median [Q1,Q3])	140 [138, 141]	140 [138, 141]	140 [138, 141]	0.013
$PaCO_2$, mmHg (median [Q1,Q3])	41 [38, 45]	41 [38, 45]	40 [37, 44]	<0.001
PaO_2, mmHg (median [Q1,Q3])	380 [311, 429]	374 [301, 425]	396.5 [335, 439]	<0.001
INR (median [Q1,Q3])	1.2 [1.1, 1.4]	1.2 [1.1, 1.4]	1.2 [1.1, 1.4]	0.008
PT, s (median [Q1,Q3])	13.8 [12.3, 15.4]	13.8 [12.4, 15.4]	13.6 [12.1, 15.3]	0.003
Scr, mg/dL (median [Q1,Q3])	0.9 [0.8, 1.1]	0.9 [0.8, 1.1]	0.9 [0.7, 1.0]	<0.001
BUN, mg/dL (median [Q1,Q3])	17 [13, 21]	17 [14, 21]	16 [13, 19]	<0.001
Type of surgery, n (%)				<0.001
CABG	1736 (48.05)	1235 (46.06)	501 (53.76)	
Valve surgery	1072 (29.67)	792 (29.54)	280 (30.04)	
Aortic surgery	16 (0.44)	14 (0.52)	2 (0.21)	
Combined surgery	114 (3.16)	84 (3.13)	30 (3.22)	
Others	675 (18.68)	556 (20.74)	119 (12.77)	

BMI, body mass index; *CHD*, coronary heart disease; *CVA*, cerebrovascular accident; *COPD*, chronic obstructive pulmonary disease; *SOFA*, Sequential Organ Failure Assessment; *SAPS II*, Simplified Acute Physiology Score II; *RBC*, red blood cell; *WBC*, white blood cell; *$PaCO_2$*, partial pressure of carbon dioxide in arterial blood; *PaO_2*, partial pressure

of oxygen in arterial blood; *INR*, international normalized ratio; *PT*, prothrombin time; *Scr*, serum creatinine; *BUN*, blood urea nitrogen; *CABG*, coronary artery bypass grafting; *CPB-AKI*, cardiopulmonary bypass-associated acute kidney injury.

Table S3 Hyperparameter settings for eight models, related to Table 2.

Model	Final Hyperparameters
Logistic Regression (GLM)	None (Default parameters)
Support Vector Machine (SVM)	sigma = 0.001, C = 0.09
Gradient Boosting Machine (GBM)	n.trees = 110, interaction.depth = 4, shrinkage = 0.1, n.minobsinnode = 25
Neural Network (NNET)	size = 4, decay = 0.4
Extreme Gradient Boosting (XGBoost)	nrounds = 10, max_depth = 3, eta = 0.001, gamma = 0.5, colsample_bytree = 0.5, min_child_weight = 1, subsample = 0.6
Adaptive Boosting (AdaBoost)	mfinal = 2, maxdepth = 2, coeflearn = "Zhu"
Light Gradient Boosting Machine (LightGBM)	objective = "binary", metric = "auc", min_data = 1, learning_rate = 1.0, nrounds = 5, early_stopping_rounds = 3
Category Boosting (CatBoost)	iterations = 100, depth = 4, learning_rate = 0.03, border_count = 32, eval_metric = "AUC"

Table S4 Primers used for qRT-PCR analysis, related to STAR Methods.

Gene	(Forward, 5'-3')	Reverse, 5'-3'
**LDHA**	GAGCCACTGTCGCCGATCTC	CCAACAGCACCAACCCCAAC
**Ccl2**	TAGCATCCACGTGCTGTCTC	CAGCCGACTCATTGGGA
**IL-1β**	CAACTGTCCCTGAACTCAACTGT	GAGATGCTGCTGTGAGATTTGAA
**TNF-α**	CAGGAGAAAGTCAGCCTCCTC	CCAGGTACATGGGCTCATACC
**β-actin**	CCCGCGAGTACAACCTTCT	CGTCATCCATGGCGAACT

Data S1: Uncropped Western Blot images

Fig. 6A

Pan-Kla

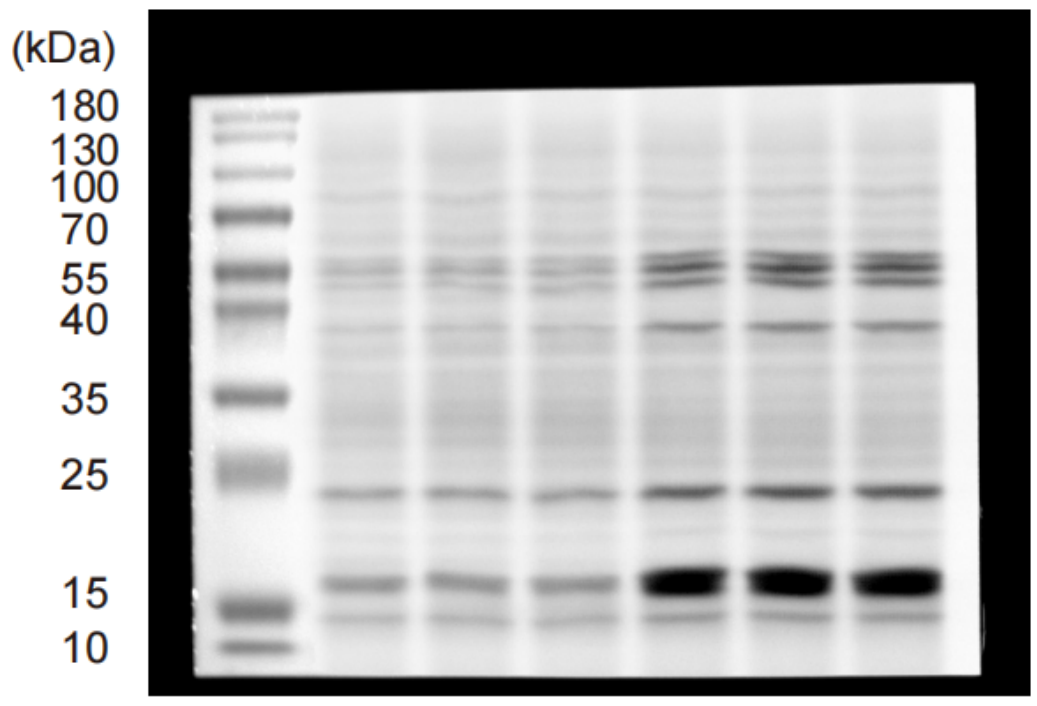

β-actin

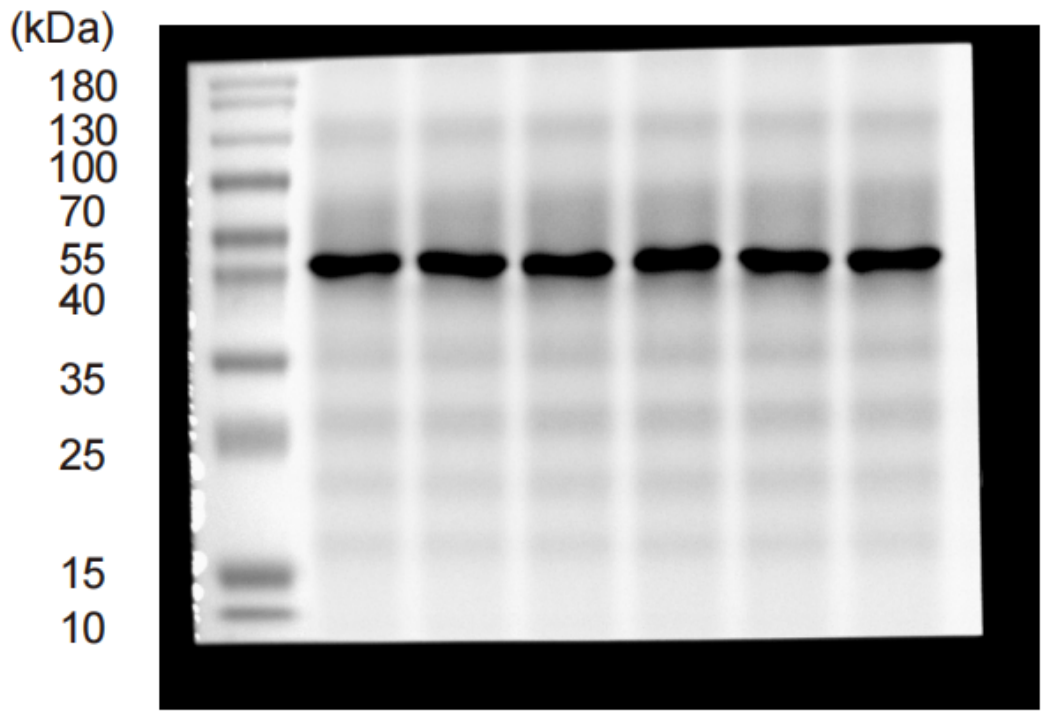

Fig. 8F

LDHA

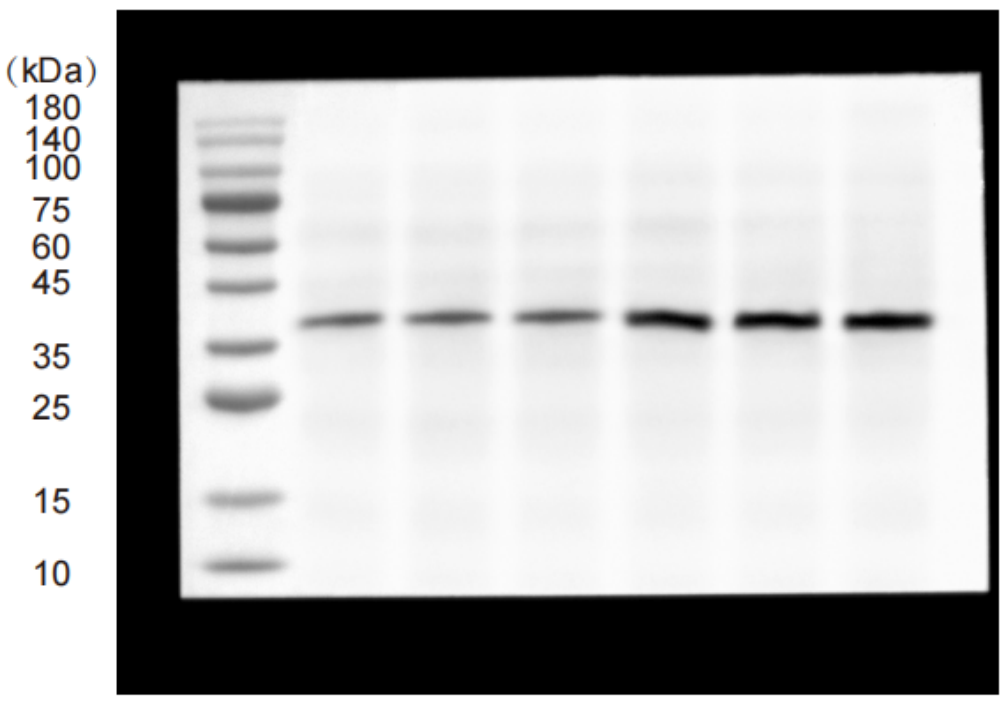

β-actin

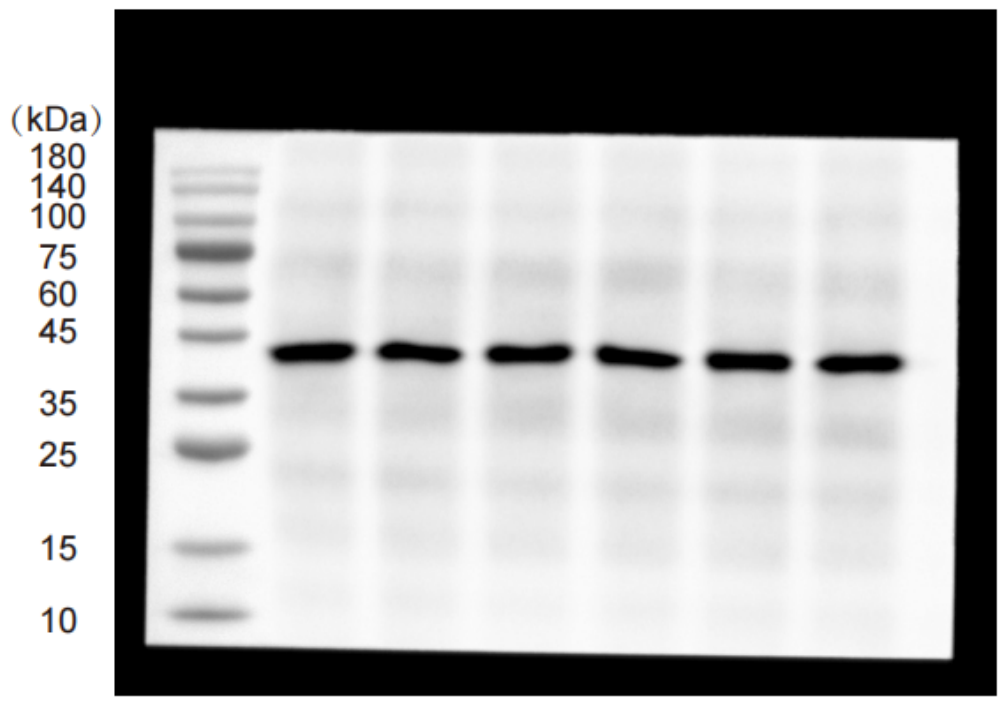

Fig. 10F

Pan-Kla

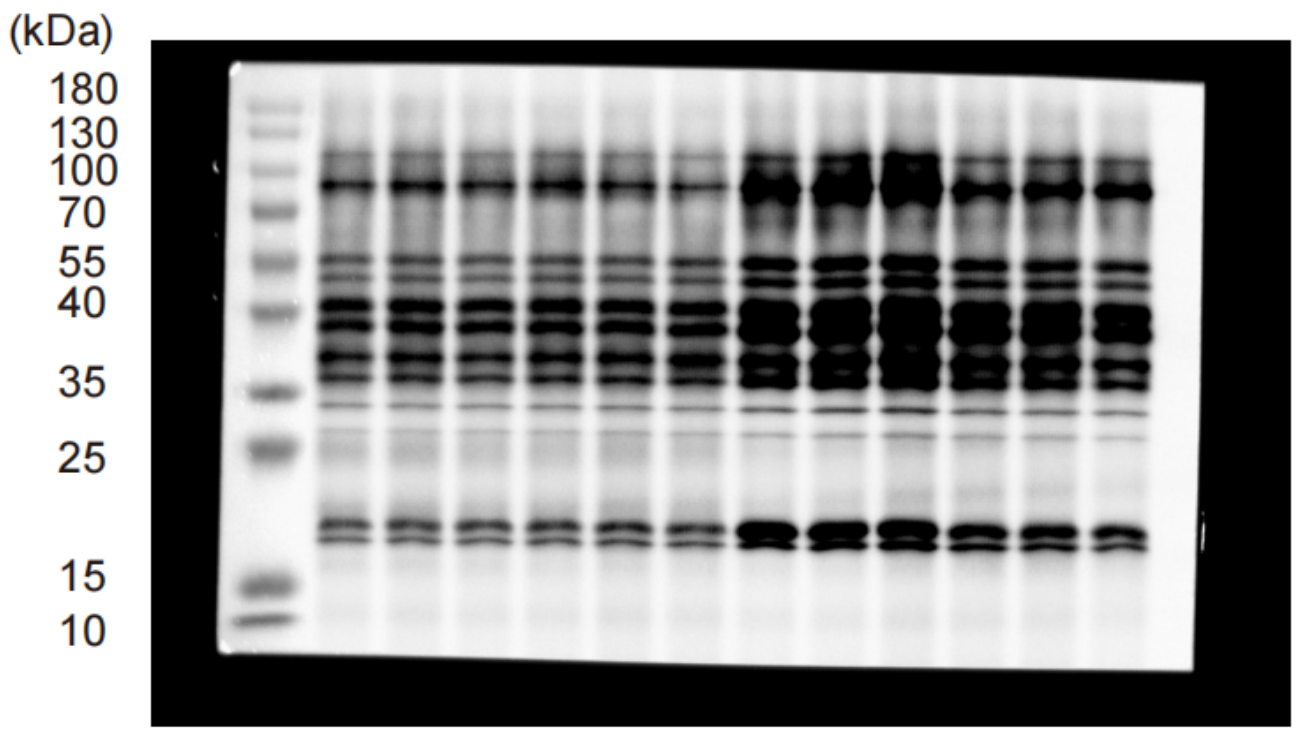

β-actin

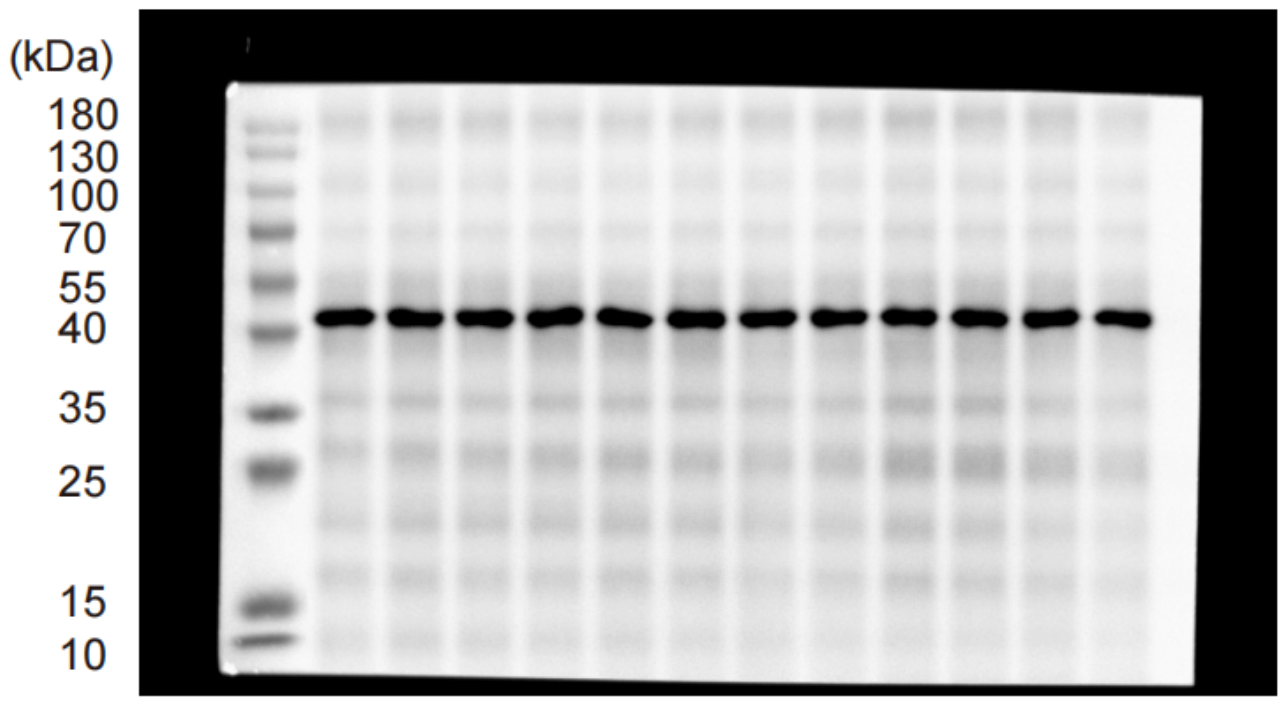

Fig. 11K

Pan-Kla

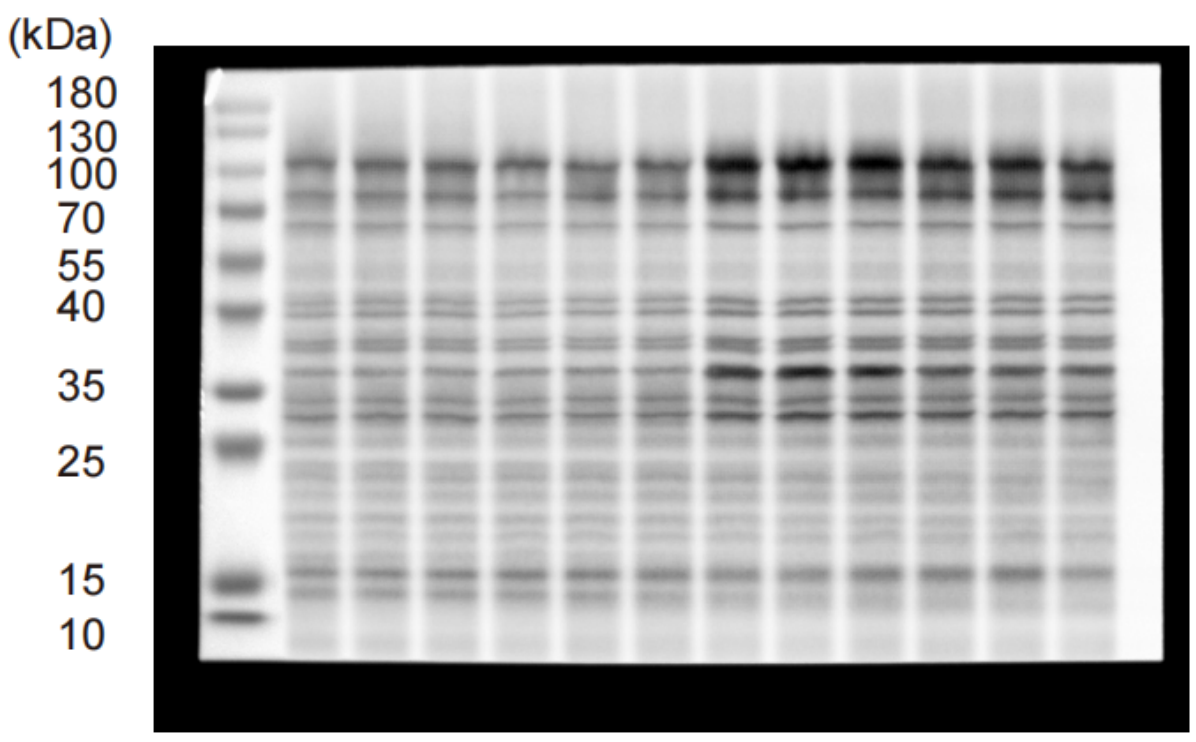

β-actin

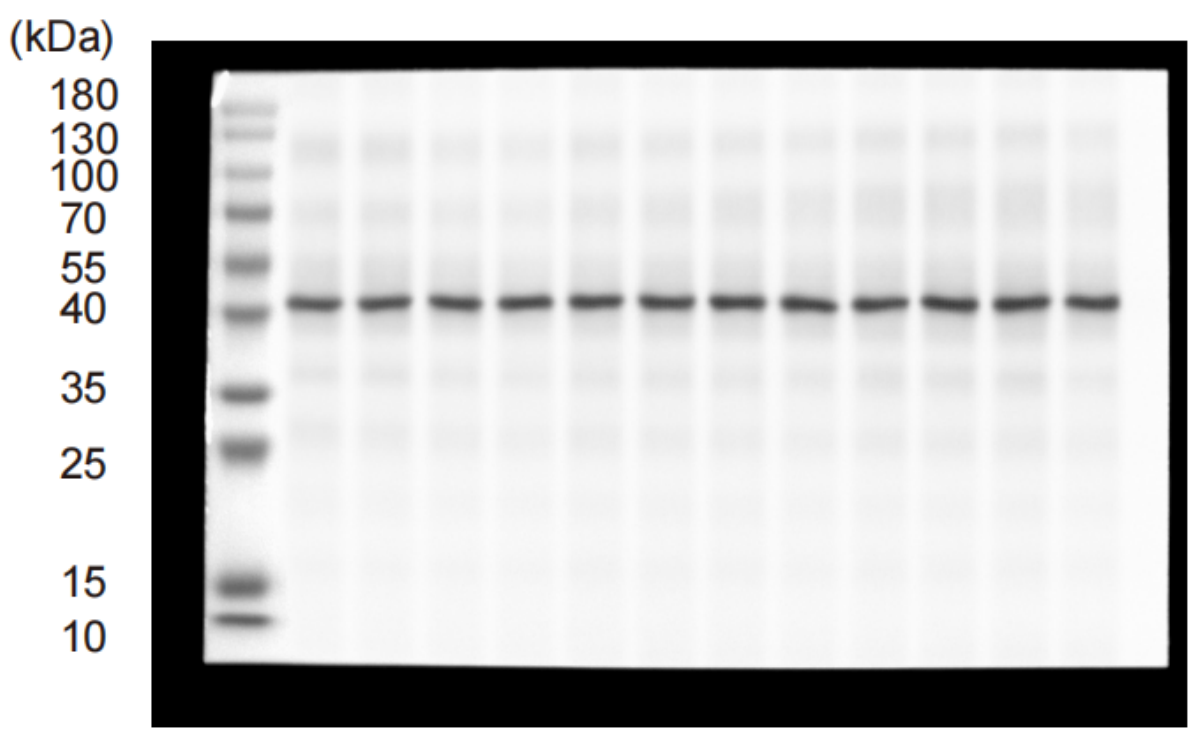

Supplement: Supplementary file 1 [file mmc1.pdf]
